# The roles of dietary lipids and lipidomics in gut-brain axis in type 2 diabetes mellitus

**DOI:** 10.1186/s12967-023-04088-5

**Published:** 2023-04-02

**Authors:** Duygu Ağagündüz, Mehmet Arif Icer, Ozge Yesildemir, Tevfik Koçak, Emine Kocyigit, Raffaele Capasso

**Affiliations:** 1 Department of Nutrition and Dietetics, Faculty of Health Sciences, Gazi University, 06490 Ankara, Turkey; 2 Department of Nutrition and Dietetics, Faculty of Health Sciences, Amasya University, 05100 Amasya, Turkey; 3 Department of Nutrition and Dietetics, Faculty of Health Sciences, Bursa Uludag University, 16059 Bursa, Turkey; 4 Department of Nutrition and Dietetics, Faculty of Health Sciences, Ordu University, 52200 Ordu, Turkey; 5 Department of Agricultural Sciences, University of Naples Federico II, Portici, 80055 Naples, Italy

**Keywords:** Lipids, Lipidomics, Type 2 diabetes mellitus, Gut microbiota, Diet

## Abstract

Type 2 diabetes mellitus (T2DM), one of the main types of Noncommunicable diseases (NCDs), is a systemic inflammatory disease characterized by dysfunctional pancreatic β-cells and/or peripheral insulin resistance, resulting in impaired glucose and lipid metabolism. Genetic, metabolic, multiple lifestyle, and sociodemographic factors are known as related to high T2DM risk. Dietary lipids and lipid metabolism are significant metabolic modulators in T2DM and T2DM-related complications. Besides, accumulated evidence suggests that altered gut microbiota which plays an important role in the metabolic health of the host contributes significantly to T2DM involving impaired or improved glucose and lipid metabolism. At this point, dietary lipids may affect host physiology and health via interaction with the gut microbiota. Besides, increasing evidence in the literature suggests that lipidomics as novel parameters detected with holistic analytical techniques have important roles in the pathogenesis and progression of T2DM, through various mechanisms of action including gut-brain axis modulation. A better understanding of the roles of some nutrients and lipidomics in T2DM through gut microbiota interactions will help develop new strategies for the prevention and treatment of T2DM. However, this issue has not yet been entirely discussed in the literature. The present review provides up-to-date knowledge on the roles of dietary lipids and lipidomics in gut-brain axis in T2DM and some nutritional strategies in T2DM considering lipids- lipidomics and gut microbiota interactions are given.

## Background

Type 2 diabetes mellitus (T2DM) is an endocrine metabolic disorder characterized by dysfunctional pancreatic β-cells and peripheral insulin resistance, resulting in abnormalities of glucose metabolism, dyslipidemia, and chronic inflammation [1]. T2DM is commonly associated with poor blood glucose control, dyslipidemia, and obesity, and has become a global public health concern due to the rising prevalence and implications of the disease [2, 3]. In 2021, about 537 million people will be diagnosed with diabetes (DM), and this number is estimated to increase to 643 million by 2030; 90–95 percent of DM diagnoses are T2DM [4, 5].

Genetic, metabolic, lifestyle, and dietary patterns are among the most influential determinants of T2DM [6]. Although ethnicity and family history/genetic predisposition have a substantial genetic basis for developing T2DM, epidemiological studies indicate that T2DM can be prevented by improving modifiable risk factors (obesity, low physical activity, and unhealthy diet) [7, 8]. In genome-wide association studies, the variants in zinc finger gene 1 (JAZF1), insulin-like growth factor 2 mRNA-binding protein 2 (IGF2BP2), transcription factor 7 like 2** (**TCF7L2), melanocortin 4 receptor (MC4R), cell division cycle 123 (CDC123), potassium voltage-gated channel subfamily Q member 1 (KCNQ1), insulin-like growth factor 2 mRNA binding protein 2 (IGF2BP2), solute carrier family 16 member 11 (SLC16A11), and PHD finger protein 2 (PHF2) have all been previously were involved in lipid metabolism and were associated with T2DM in adults [9, 10].

Dyslipidemia, caused by elevated plasma triglyceride (TG), increased low density lipoprotein cholesterol (LDL), and reduced high dense lipoprotein cholesterol (HDL), is typically observed in T2DM [11]. Mitochondrial dysfunction, ER stress, inflammation, abnormal fatty acid regulation, and β-oxidation may all be involved in dyslipidemia [12, 13]. Excessive dietary intake of free fatty acids (FFA) in patients with T2DM induces the synthesis of phospholipids, glycerolipids, and sphingolipids, hence exacerbating insulin resistance and resulting in lipotoxicity [14, 15]. Currently, lipidomics will help in the understanding of changes in lipid metabolism and elucidate the metabolic pathways underlying the relationship between diet, dyslipidemia, and T2DM.

Lipidomics is a branch of metabolomics that identifies and quantifies the lipids produced by cells in response to pathogenic stimuli [16]. The purpose of lipidomics research is to identify and quantitatively determine the spectrum of intact lipid molecules found in cells and biological fluids, as well as to relate their composition to genetics, proteomics, nutrition, and disease [16, 17]. Recently, increased lipidomic research has been conducted to identify biomarkers for and explain the cellular pathogenic processes of lipid metabolism abnormalities, which play a role in the pathogenesis of several diseases including T2DM, cancer, Alzheimer's disease, cardiovascular disease, nonalcoholic fatty liver disease, and obesity [18–22]. Since the 2000s, lipidomics has been used to identify potential predictive and diagnostic parameters of T2DM [23, 24].

Population-based studies have indicated that phospholipids containing glycine, lysophosphatidylcholine acyl, acetylcarnitine, α-hydroxybutyrate, and choline are precursors of glucose tolerance abnormalities. Due to the lack of information on plasma lipidome changes during the shift from prediabetes to T2DM, the plasma lipid that may be used to diagnose T2DM has not been precisely determined. These molecules can influence the interaction along the gut-brain axis, hence contributing to the pathogenesis of T2DM. Furthermore, it has been shown that some lipids such as triglycerides and sphingolipids modulate insulin resistance and T2DM. [25–28]. The current review summarizes the effect of dietary fats and lipidomics on the gut-brain axis, the role of lipidomics, and possible mechanisms of action in T2DM.

## Dietary lipids

Dietary fats are divided into three subgroups: unsaturated FA, saturated fatty acids (SFA), and trans fatty acids (TFA). Unsaturated FA consists of monounsaturated fatty acids (MUFA) and polyunsaturated fatty acids (PUFA) [29]. Although the fatty acid composition of the diet plays a significant role in enhancing insulin sensitivity and reducing T2DM and T2DM-related complications, the underlying mechanisms remain unclear. In terms of dietary fat, the Food and Agriculture Organization of the United Nations (FAO) concluded in 2010 that a high dietary intake of SFA is a "possible" risk factor for T2DM, while a high intake of TFA is also a "possible" risk factor for T2DM. It has been claimed that PUFA intake was a "possible" positive effect on T2DM, whereas the available evidence for MUFA was insufficient [30]. The widespread view is that the fatty acid composition of the diet might alter cell membrane function [31]. The fatty acid content of the cell membrane regulates several physiological systems, including membrane fluidity, cellular activities, ion permeability, insulin receptor affinity, translocation of glucose transporters interacting with second messengers, and membrane fluidity. All of these alterations can alter the tissue and organ insulin sensitivity [32].

The literature has shown the processes through which FAs exert a direct regulatory influence on gene expression and enzyme function [33]. In vitro*,* while PUFA (arachidonic acid (AA) > eicosapentaenoic acid (EPA) > docosahexaenoic acid (DHA) > linoleic acid (LA)) activates nuclear receptors, including peroxisome proliferator-activated receptor (PPAR), SFA and MUFA have only a minimal influence on lipogenic gene expression [34, 35]. Furthermore, SFA alters glucose metabolism by regulating inflammatory gene expression, transcription factor activity, and enzyme activity [36]. LA prevents DM through its anti-inflammatory properties. Omega 3 and omega 6 FAs rich in diet can increase insulin sensitivity by suppressing hepatic lipogenesis and promoting FAs oxidation [37].

Many foods contain SFA, but only in low amounts; those of animal origin, including milk, butter, cheese, and meat, are the main sources of SFA in the typical diet. Exceptions include fats derived from tropical plants, which are usually low in SFA but high in the palm and coconut oil. Lauric acid (C12:0), myristic acid (C14:0), palmitic acid, and stearic acid (C18:0) are the majority of dietary SFAs [38]. Palmitic acid and foods derived from animals induce inflammation, oxidative stress, and inflict irreversible harm to cardiometabolic health by interfering with nitric oxide and insulin signaling [39, 40].

A high dietary intake of SFA adversely impacts glucose and lipid metabolism by increasing hyperglycemia, hyperinsulinemia, and insulin resistance in metabolic organs such as the liver, pancreas, adipose tissue, and kidney. A diet rich in SFAs decreases the number of large islets in the pancreas, resulting in a more intense insulin response to a glucose load. The structure and function of the islets are altered, resulting in glucose sensitivity and T2DM [41, 42]. It impacts lipid homeostasis, differentiation of adipocytes, fat cell volume and number, and promotes weight gain by increasing the white adipose tissue (WAT) in adipose tissue. All of these alterations accelerate the development of inflammation and leukocyte infiltration in adipose tissue. The accumulation of hepatic triacylglycerol, glucose intolerance, elevated blood sugar, and elevated insulin levels are all consequences of this condition. Increased blood lipid concentrations result in insulin resistance in peripheral tissues, impaired glucose absorption and usage and lipid metabolism, blood circulation and lipid aggregation in various tissues, and metabolic signaling pathways that regulate insulin secretion in pancreatic β-cells. Maintaining the homeostasis of the organism requires tight regulation of glucose and lipid catabolism [43–45].

Epidemiological, case–control, and prospective cohort studies have demonstrated that an increase in dietary SFA consumption is associated with the development of T2DM [46–48]. Short-term intervention studies indicate that a high SFA diet raises insulin, fasting blood glucose, and Hba1c levels relative to a high MUFA or PUFA diet [49, 50]. However, there are other research suggests that there is no relationship between SFA intake and the onset of T2DM [51–53]. It is considered that dietary SFAs influence glucose metabolism and T2DM by inducing insulin resistance.

Trans fatty acids (TFA) are found in ruminant milk and meats, as well as in partially hydrogenated vegetable oils [54]. Some of the metabolic impacts of TFA include an increase in obesity, and insulin resistance, inflammation, and a reduction in endothelial function. TG and lipoprotein (a) levels are increased, whereas total cholesterol (TC):HDL ratio, apoB: apoA ratio, and TC: HDL ratio are all decreased by TFAs [55, 56]. TFA inhibits the antilipolytic impact of insulin in adipose tissue and insulin-mediated glucose transport in animal models. Resistin reduces insulin sensitivity by increasing messenger RNA (mRNA) expression and decreasing PPAR and lipoprotein lipase expression [57, 58].

Olive oil, canola oil, and some plant oils are among the richest sources of MUFA in the diet, whereas red meat, milk, and dairy products also contain MUFA. Olive oil, which is rich in oleic acid (C18:1n-9), and erucic acid (C22:1n-9) are the two most prevalent dietary sources of MUFA in regions where rapeseed oil consumption is prominent [38]. Oleic acid enhances FA oxidation by protein kinase A (PKA)-mediated deacetylation of the Sirtuin1 (SIRT1)-peroxisome proliferator-activated receptor co-activator-1α (PGC1α) complex. Oleic acid exerts an anti-inflammatory effect by participating in several metabolic pathways. Upregulation of M2 expression, the elevation of adiponectin level, the diminution of phosphate and tensin homolog, downregulation of protein phosphatase 2A, and induction of macrophage polarization are all effects of E-selectin, soluble intercellular adhesion molecule-1 (ICAM-1), interleukin (IL)-6, and tumor necrosis factor alpha (TNF-α). Additionally, oleic acid regulates insulin resistance and T2DM by lowering glucolipotoxicity, oxidative stress, and enhancing β-cell function and endothelial and hypothalamus function [45, 59, 60]. A diet that is rich in MUFA increases insulin sensitivity by lowering the glycemic load and insulin requirement. Through an increase in the number of hepatic LDL receptors, it accelerates the turnover of LDL cholesterol. Many beneficial compounds, including phenolic compounds, phytochemicals, and fat-soluble vitamins, are concentrated in foods high in MUFA [61–63].

LA and alpha-linoleic acid (ALA) are the only types of necessary FAs that may be obtained from food due to the absence of desaturase enzymes in the human body [64]. Both LA, which is the precursor of omega 6 FAs, and ALA, which is the precursor of omega 3 FAs, are converted to other PUFAs through the addition of double bonds and acyl chains by the enzymes desaturase and elongases, respectively [65, 66]. Omega 3 and omega 6 are both types of FAs that are found in PUFA. Omega 3 FAs consist of long-chain EPA and DHA, both of which are often found in fish, and ALA, which is present in some plant oils such as flaxseed, rapeseed, and canola oil. Canola oil, soybean oil, corn oil, and sunflower oil are just a few of the various plant oils rich in omega 6, particularly LA and dihomo γ LA [67].

Gene expression, the metabolism of prostaglandin and leukotriene, and the synthesis of interleukin (IL)-1 are all significantly impacted by the ratio of omega 6 to omega 3 FAs that exist in the organism under physiological conditions. Omega 3 and omega 6 FAs are in constant competition for desaturation enzymes. In enzymatic processes, fatty acid desaturases 1 (FADS1) and 2 (FADS2) prefer ALA to LA.

A disruption in the ratio of omega 6 and omega 3 initiates a prothrombotic and proinflammatory process that favors omega 6 in the organism and leads to the development of atherosclerosis, obesity, and DM [68, 69]. Long-chain PUFAs improve adipocyte membrane fluidity, GLUT4 RNA and protein levels, hence increasing the number of insulin receptors. Insulin stimulates the activities of desaturases 5 and 6. This increases the amount of insulin receptors on the cell membrane and the affinity of insulin for its receptor, so enhancing the effects of insulin on the organism. However, in the presence of an excess of omega 6 and a deficiency of omega 3, external stimuli release AA from the cell membrane and the formation of proinflammatory mediators [70]. It has been reported that the ratio of omega 6 to omega 3 in the diet should be 1:1 or 2:1 for optimal health [71].

Omega 3 FAs improve insulin sensitivity by reducing ER stress in mitochondria and enhancing the β-oxidation of FAs, thus decreasing the accumulation of lipids and reactive oxygen species (ROS) [59]. Additionally, omega-3 FAs have a beneficial impact on mitofusin 2, a protein implicated in mitochondrial dynamics, homeostasis, and the preservation of the membrane integrity of mitochondria [72]. EPA and DHA modulate insulin sensitivity via Akt phosphorylation, AMP-activated protein kinase, and activating PPARγ [73]. Also, omega-3 FAs regulate pancreatic β-cell insulin secretion by acting on the function and structure of lipid rafts and indirectly reducing the development of proinflammatory mediators in adipose tissue and increasing adipokine synthesis. Omega 3 FAs inhibit inflammatory cytokines, induce adipose tissue to produce adipokines, and increase insulin secretion by directly influencing β-cell activity by binding to PPARs, G protein-coupled receptor 40 (GPR40), and GPR120. PUFA binds to GPR120 in adipose tissue, resulting in increased GLUT4 translocation and glucose uptake in adipose tissue [74, 75].

In randomized controlled trials examining the impact of omega 3 FAs on glycemic control [EPA (2 g/day, 95% pure EPA), fish oil per day (2 g/day EPA + DHA), omega 3 FAs (1.6 g/day EPA and 0.8 g/day DHA)], fasting plasma glucose, HbA1c, and HOMA-IR were reported to be reduced [76–78]. Prospective cohort studies have shown that consuming lean fish reduces the incidence of T2DM. It has been observed that consuming lean seafood and fish improves insulin sensitivity and lowers insulin resistance in individuals with insulin resistance [79–82]. Studies have revealed that EPA and DHA play a significant part in lowering the risk of lipotoxicity and preserving insulin sensitivity [83–85]. In several investigations, there was no significant correlation between omega 3 FAs and glycemic indicators such as fasting plasma glucose, insulin, and HbA1c [86–88].

There was not enough evidence on the relationship between dietary omega 6 intake, desaturase enzymes, and the incidence of T2DM [67]. In dietary intervention and prospective cohort studies using omega 6 biomarkers/food consumption frequency forms, however, an inverse association was observed between LA and T2DM prevalence [40, 89]. It has been determined that whereas high levels of γ-LA and Di-homo-γ-LA increase the risk of T2DM, high levels of LA diminish this risk. According to research findings, omega 6 may be an indicator of hyperinsulinemia rather than a risk or protective factor for T2DM [90, 91]. In intervention studies that replaced dietary SFA with MUFA, one research revealed that insulin sensitivity was enhanced [92], Other studies have shown that fasting insulin and insulin sensitivity are unchanged [93, 94]. Table 1 shows the classification and nutritional importance of dietary lipids in T2DM. To sum up, it was shown that hepatic fat deposition was reduced and insulin sensitivity increased in interventions involving the consumption of omega 6 instead of dietary SFA [95, 96].

**Table 1:** Classification and nutritional importance of dietary lipids in T2DM

Type of fat	Description	Dietary sources	Role of mechanism
Saturated fatty acids [34, 37, 41, 43, 49]	Single-bond fatty acids, such as lauric acid, palmitic acid, stearic acid	Meat and sausages, butter, milk and dairy products, palm oil, coconut oil	*In the pancreas, liver, adipose tissue* Increase inflammation, endoplasmic reticulum stress, lipid oxidation, reactive oxygen species, and lipotoxicity Decrease β-cell function and mass, insulin production, insulin receptor expression and activity, mitochondrial function, adiponectin secretion, and insulin-induced glycogen synthesis *In muscle* Induce mitochondrial dysfunction, SFA-induced insulin resistance, and impaire glucose metabolism Reduce insulin signaling *In gut* Increase in local and systemic LPS concentrations, systemic inflammation, and metabolic endotoxemia
Trans fatty acids [34, 45, 52, 54]	Polyunsaturated fatty acids whose double bonds are in the "trans" position Industrially (hydrogenation), excessive heating of oil with a high level of unsaturated fatty acids naturally produce by ruminants	Margarine, butter, milk and dairy products, meat	*In the pancreas, liver, adipose tissue* Increase in oxidative stress, inflammation, and insulin resistance Decrease in insulin secretion Impare β-cell and ER function, glucose uptake *In muscle* Increase oxidative stress Impare glucose uptake and insulin-stimulated glucose metabolism *In gut* Increased inflammation and metabolic endotoxemia Cause lipotoxicity
Monounsaturated fatty acids [34, 45, 57, 59]	Fatty acids with one double bond in the fatty acid chain (cis-position) such as oleic acid and palmitoleic acid	Plant-based food: olive oil, safflower oil, hazelnuts, almonds, pistachios, olives, avocados Animal-based foods: eggs, butter, dairy products, meat and meat products	*In the pancreas, liver, adipose tissue* Improve fasting glucose, insulin resistance, fasting glucose, and β cell function, increase GLP-1, adiponectin levels, FA oxidation, activate PPARγ receptors Decrease inflammation *In muscle* Increase membrane translocation of GLUT 4 and glucose uptake *In gut* Improve gut homeostasis and decreases gut dysbiosis
Polyunsaturated fatty acids [32–34, 65]	Fatty acids with more than one double bond in the fatty acid chain (cis-position) such as omega 6 fatty acids and omega 3 fatty acids	Dietary sources of omega 6 and omega 3 fatty acids	
Omega 3 fatty acids [32, 33, 58, 73, 76]	Polyunsaturated fatty acids with the last double bond in the omega 3 position (the third position counting from the methyl end) such as ALA, EPA, DHA *EPA and DHA can be synthesized in the body from ALA	ALA is an important molecule found mostly in plant-based foods such as rapeseed oil, linseed oil, soybean oil, and walnut EPA and DHA are mostly found in foods derived from animals such as salmon, tuna, and microalgae	*In the pancreas, liver, adipose tissue* Increase insulin secretion, FA oxidation, PPARγ activity, glucose uptake via GLUT-4 translocation, and mitochondrial biogenesis Improve insulin resistance and insulin secretion Decrease ER stress, inflammation, and lipogenesis *In muscle* Increase in FA oxidation, insulin action on glucose usage, and protein synthesis Decrease inflammation *In gut* Increase in insulin sensitivity through GPR120 Decrease in metabolic endotoxemia, insulin resistance
Omega 6 fatty acids [32, 33, 84, 87, 89]	Polyunsaturated fatty acids with the last double bond in the omega 6 position (the sixth position counting from the methyl end) such as LA, AA *Omega 6 fatty acids can be synthesized in the body from AA	Sunflower oil, corn oil, meat, butter, milk and dairy products, egg yolk	*In the pancreas, liver, adipose tissue* Improve insulin sensitivity, inflammatory cytokines Stimulate FA oxidation Inhibit lipogenesis *In muscle* Enhance affinity for the FA transporters *In gut* Increase inflammation

## Molecular regulations, and homeostasis of dietary lipids in T2DM

T2DM as a systemic disease as is characterized by hyperinsulinemia, insulin resistance, and relative insulin deficiency [6]. The pathogenesis of diabetic complications involves genetic and epigenetic changes, dietary factors, and a sedentary lifestyle [97, 98].

The metabolism of proteins, lipids, and glucose are all significantly regulated by insulin. It participates in the regulation of glucose uptake in muscle, adipose tissue lipolysis, and muscle proteolysis, as well as the metabolism of hepatic glucose and triglycerides [99]. The regulation of lipid metabolism is impacted by increased oxidative and ER stress, hyperglycemia, lipidemia, insulin resistance, and impaired pancreatic beta-cell function in T2DM [100]. Lipid metabolism includes both the biosynthesis and breakdown of lipids like cholesterol, triglycerides, and FAs. Specialized lipoproteins carry lipids from the intestine to the liver (where most lipid conversion occurs) and from the liver to peripheral tissues [101]. Dyslipidemia, T2DM, and obesity are all linked to abnormalities in lipid metabolism, which frequently result in metabolic complications like insulin resistance, DM, ectopic lipid accumulation, non-alcoholic fatty liver disease, and atherosclerosis [102]. The regulation of lipid metabolism is greatly influenced by nutrients, particularly sugars and FAs [103]. Triglycerides account for 90.0% of all dietary lipids (TG). Phospholipids are also present in cholesterol and cholesterol esters in the diet, in addition to triglycerides [104]. The lingual lipase enzyme and gastric lipase enzymes in the mouth and stomach emulsify the digestion of dietary lipids in adults, but digestion hardly ever occurs. Chyme, a form of lipid, enters the small intestine [105, 106]. The mixture, known as Chyme, causes intestinal cells to secrete the hormone secretin. Secretin facilitates the breakdown of lipids by encouraging the pancreas to secrete bicarbonate, pancreatic lipase, cholesterol esterase, and phospholipase A2. Additionally, cholecystokinin increases bile secretion by promoting gallbladder contraction. Lipids are emulsified in the duodenum with bile salts released from the gallbladder, increasing the surface area of the lipid droplet and improving the efficiency of digestive enzymes [107]. Non-esterified fatty acids (NEFA), CD36, and Niemann-Pick C1-like 1 protein (NPC1L1) for cholesterol are three specific transporters that enable enterocytes to absorb dietary lipids. Triacylglycerols (triglycerides), cholesteryl esters, and other lipids (phospholipids and trace amounts of unesterified cholesterol) come together in enterocytes to form chylomicrons, which are then combined with apolipoprotein (Apo)B-48 (also ApoA-IV and ApoA-I) [108]. Liquid triglycerides make up 90% of the chylomicron mass. These chylomicrons travel from the intestinal mucosa to the lymphatic system via exocytosis, and the large chylomicrons travel from the blood to the liver and adipose tissue [109]. In particular, lipoprotein lipase (LPL) mediates intravascular lipolysis of triglyceride-rich lipoproteins (TGs in chylomicrons are broken down into FFA and glycerol) and forms chylomicron remnants, which are important for lipid homeostasis and chylomicron clearance [110]. Insulin increases the amount of LPL mRNA, which stimulates LPL. As a result, they control apoC-II and LPL activities in concert through both transcriptional and post-translational mechanisms [111]. The liver cells' ApoE receptors can detect chylomicron remnants. Chylomicrons are more likely to bind to heparan sulfate proteoglycans (HSPGs) on hepatocyte surfaces and be taken up into the liver by LDL receptor-associated protein (LRP) or LDL-R when ApoE is present. In the process of removing lipoprotein residues, apoE is stored in heparan sulfate proteoglycans. In apoB-48, the ligand binding site is missing [112]. Insulin increases the uptake and clearance of chylomicron residues by inducing the translocation of lipoprotein receptor-related protein to the plasma membrane [113]. By increasing the expression and activity of LDL-receptors, it also encourages LDL clearance [114]. When the 125I-labeled activated α2M (α2M ∗) pathway is activated by insulin secretion increases LRP and LDL-R's function by 2–3 times. Insulin increased the liver's LRP-specific uptake of chylomicron residues, according to a rat study. Postprandial lipoprotein metabolism may suffer if insulin-mediated signaling pathways are disrupted [115]. In HepG2 cells, insulin forms heterodimers with ER small subunit protein disulfide Isomerase to catalyze the transfer of lipid to nascent apoB, a rate-limiting step in the production of hepatic very low-density lipoprotein (VLDL). Additionally, insulin controls the hepatic microsomal triglyceride transfer protein (MTP) metabolism by separating the complex formed by the mitogen-activated protein kinase (MAPK) pathway, Foxa2, which is phosphorylated in response to insulin action, and PGC-1β [116]. In this study, it was determined that subjects who were insulin resistant had excessive VLDL production and unrestricted MTP expression [117].

The free cholesterol that HDL absorbs from tissues is esterified using lecithin cholesterol acyl transferase (LCAT). Free cholesterol is quickly esterified by LCAT after being absorbed by HDL. They serve as an Apo C and Apo E circulating store for HDL, VLDL, and chylomicrons. They use scavenger receptor B1 (SR-B1) receptors to remove and esterify free cholesterol from extra hepatic tissues and transport it to the liver [118]. Small size HDL (commonly known as HDL3) grows via ester transfer (usually called HDL2) [119]. Also, HDL demonstrates anti-inflammatory, antioxidant, anti-thrombotic, and anti-apoptotic properties [120]. Additionally, VLDL and LDL can exchange lipids via the cholesteryl ester transfer protein (CETP) provided by HDL. In this manner, triacylglycerols are reciprocally transferred from VLDL to HDL and cholesterol esters from HDL to VLDL [121]. The physiological control of HDL cholesterol metabolism depends on insulin. It directly affects the liver, encouraging the transition from HDL2 to HDL3 (through its action on hepatic lipase) [122].

**Fig. 1:**
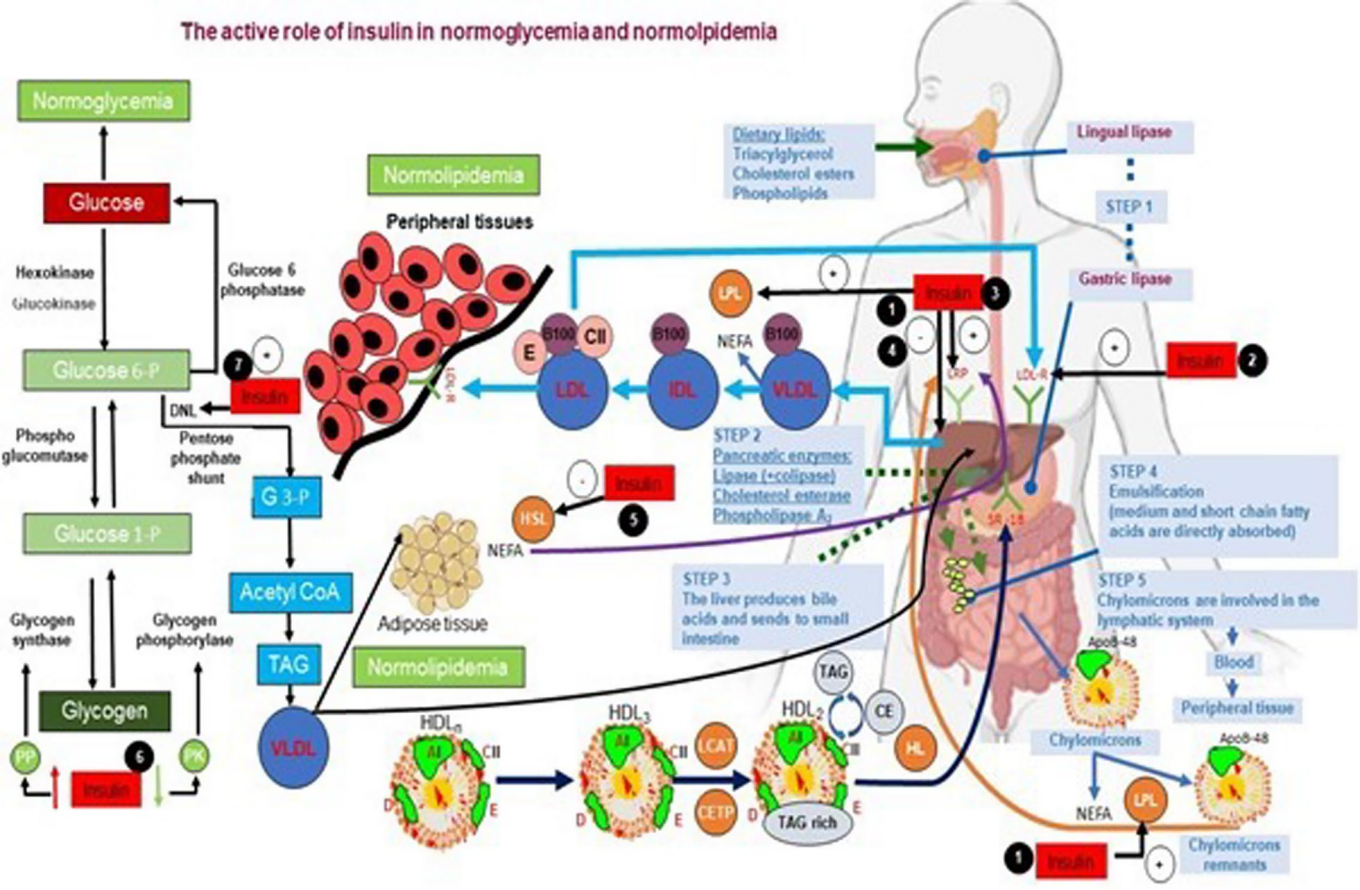
The active role of insulin in normoglycemia and normolipidemia. An overview of Interaction between impared glucose and lipids metabolisim inT2DM. (1) increased chylomicron production, (2) reduced catabolism of both chylomicrons and VLDLs (diminished LPL activity), (3)increased VLDL production (mostly VLDL1), (4) reduced LDL turnover (5) increased production of large VLDL (VLDL1) preferentially taken up by macrophages; LDL (qualitative and kinetic abnormalities): (6) low plasma adiponectin favouring the increase in HDL catabolism. (7) increased number of glycated LDLs, small, dense LDLs (TAG-rich) and oxidised LDLs, which are preferentially taken up by macrophages; (8) increased CETP activity (increased transfer of triacylglycerols from TAG-rich lipoproteins to LDLs and HDLs), (9) increased TAG content of HDLs, promoting HL activity and HDL catabolism, (10) İmpaired glucose metabolisim. (11) İmpaired de novo lipid metabolisim (Acetyl CoA and NADPH inhibit pyruvate dehydrogenase as a result of B oxidation. The lactate and alalnin thus formed increase hyperglycemia because of gluconeogenesis (ketone bodies formation increases) in the liver.) *CE* cholesterol ester, *CETP* cholesteryl ester transfer protein, *HDLn* nascent HDL, *HL* hepatic lipase, *HSL* hormone-sensitive lipase, *LPL* lipoprotein lipase, *SR-B1* scavenger receptor B1, *TAG* triacylglycerol, *PP* protein phosphatase, *PK* protein kinase, *NEFA* non-esterified fatty acids *DNL*: de novo lipogenesis, *LCAT* Lesitin-kolesterol acil transferaz, *G3P* gliserol 3-fosfat protein kinase

Circulating carbohydrates are transformed into FAs through the intricate process known as de novo lipogenesis (DNL), which is then used to create triglycerides or other lipid molecules. The primary carbon source for the synthesis of FAs comes from the glucose metabolites created during glycolysis [123]. After consuming a large of carbohydrates, circulating glucose is absorbed by adipocytes via insulin-stimulated GLUT4, where it is converted to pyruvate by glycolysis in the cytosol and then transported to the mitochondria for further oxidation in the tricarboxylic acid cycle (TCA). In the cytosol, novo lipogenesis uses citrate, an intermediate of the TCA cycle, as a substrate. Adipocyte lipogenesis is significantly regulated at the transcriptional level by the protein carbohydrate response element-binding protein (ChREBP). In addition to activating ATP-citrate lyase (ACLY), acetyl-CoA carboxylases 1 (ACC1), fatty acid synthase (FASN), and stearoyl-CoA desaturase-1 (SCD1), insulin activates Max-like protein X (MLX) and ChREBP-, which in turn promote the expression of target genes that support the synthesis of FAs [123]. Disruptions in lipid and glucose metabolism and a decline in hepatic glycogen synthesis are brought on by problems with insulin secretion and insulin resistance. Ectopic lipid-induced muscle insulin resistance comes first, followed by liver insulin resistance, which directs ingested glucose to the liver, increasing hepatic de novo lipogenesis and hyperlipidemia [124]. The active role of insulin in normoglycemia and normolpidemia is presented in Fig. 1.

The metabolism of glucose and lipids are interconnected in numerous ways. Diabetic dyslipidemia, which is characterized by elevated triglycerides, low HDL-C, and a predominance of small-dense LDL particles, is the most significant clinical manifestation of this interaction [125]. Triglyceride-rich lipoproteins (TRLs) tend to accumulate more frequently in diabetic dyslipidemia [126]. The combination of a defect, excessive production of TRL-apoB-100, also known as very low-density lipoprotein (VLDL, primarily VLDL1) from the liver, and removal of TRL-apoB-48, also known as chylomicrons, from the gut results in diabetic dyslipidemia [127]. Phosphatidylinositol 4,5-biphosphate (PIP2) is converted to phosphatidylinositol 3,4,5-triphosphate (PIP3) when insulin binds to its receptor, which causes tyrosine phosphorylation, activation of PI3K, and other processes (PIP3). Serine/threonine kinase Akt is activated by PI3K activation, which also inhibits the insulin-mediated inhibition of phospholipase D1 and ARF-1, two components involved in the formation of VLDL1, and decreases the synthesis of ApoB [128]. Both TNF-receptor 1 (TNFR1) and TNF-receptor 2 (TNFR2), as well as the Src homology 2 domain containing RS-1, Akt S473, and T308, and the (Shc) adapter, are less phosphorylated and more insulin resistant because of TNFα induction [129]. Impaired insulin metabolism results in dyslipidemia, which is accompanied by increased apoB stability, MTP, and TNFα release [130]. LPL activity is increased by insulin and decreased by insulin resistance. Chylomicron levels are typically higher in T2DMs with insulin resistance because of both chylomicron overproduction and decreased catabolism. Additionally, through the activation of FOXO1, insulin resistance promotes the production of apoC3, an inhibitor of LPL. The decreased VLDL catabolism is impacted by rising apoC3 serum levels and their impact on LPL metabolism [131].

The treatment of disorders of lipid and insulin metabolism depends on maintaining the ideal dietary balance. Oleic acid consumption that is adequate and well-balanced lowers leukotriene B4 levels and boosts insulin sensitivity, improving insulin sensitivity [60]. LDL-R activity declines and LDL turnover deterioration because of disruption of insulin metabolism. A decline in LDL B/E receptors may be the cause of impaired LDL catabolism (LDL receptor capable of binding apoB and apoE) [132]. In a study, insulin plays a significant role in the expression of LDL-R in vivo and that, in patients with T2DM and poor metabolic control compared with non-diabetic patients, LDL-R expression decreases despite oral anti-diabetic therapy and returns to normal after 3 months of insulin therapy [133]. While macrophages are essential for preserving normal tissue homeostasis, they also play a significant role in the emergence of low-grade inflammation. Insulin resistance results from low-grade inflammation, and low-grade inflammation develops and progresses because of both insulin resistance and hyperinsulinemia [134, 135]. The activation of macrophages with the IL-4 and interferon-γ (INFγ) signaling pathways is influenced by insulin-activated IRS-MAPK-PI3K and its modulating protein kinase B (PKB)/Akt kinase pathway [136]. IL-1, IL-2, IL-4, IL-5, IL-6, IL-12, IFN-γ, TNF-α, IL-10, and lipopolysaccharide are other proinflammatory cytokines that are linked to increased FoxO1 activity brought on by impaired insulin metabolism. It also influences how macrophages are stimulated [137]. There is various oxidation levels in LDL particles in people with T2DM, from minimally oxidized LDL (MM-LDL) to fully oxidized LDL (Ox-LDL). Due to its negative effects on -cells, an elevated Ox-LDL concentration is also linked, and this creates a vicious cycle, to an increased risk of DM. Initial oxidative changes in LDL lipids occur when apoB-100, also referred to as MM-LDL, is not present. LDL lipids undergo oxidation at a later stage, where they turn cytotoxic and proapoptotic [138]. Because of impaired insulin metabolism, induced regulation of glycated and oxidized LDLs (PI3K/PKB/PPARγ) results in the formation of lipid peroxidation products, cytokine release, ROS production, and inflammation [139, 140]. With their hypolipidemic effect, omega 3 FAs in the diet control how VLDLs are formed in the liver and lower lipogenesis by releasing less TAG [141]. EPA and DHA acids, two long chain omega 3 PUFA, are beneficial for controlling lipid metabolism. apo B is degraded and fatty acid β-oxidation is increased, while TRL apoB-48 secretion and diacylglycerol acyltransferase, fatty acid synthase, and de novo lipogenesis are inhibited, increased, and acetyl CoA carboxylase is carboxylated, respectively, by omega 3 PUFAs [142]. A randomized controlled study found that supplementing with -3 PUFAs (4 g/day, 46% EPA and 38% DHA) significantly decreased the release of TRL apoB-48 [143]. Another randomized controlled trial found that in people with impaired glucose regulation, omega 3 FAs or their combination (2-g fish oil (1000-mg EPA + 400 mg DHA)) significantly reduced inflammation, reduced insulin resistance, and improved glucose and lipid metabolism [144]. Through the inhibition of DNL, an increase in fatty acid oxidation, and a reduction in ApoB synthesis, dietary omega 3 PUFAs prevent the secretion of VLDL [[145]. Additionally, omega 3 PUFAs reduces the production of the proinflammatory cytokines IL-1, IL-6, TNF-α, and TNF- β in response to an inflammatory stimulus, indirectly regulating lipid metabolism [146]. Because they bind to PPARs, GPR40, and GPR120, omega 3 PUFAs have a direct impact on -cell function and increase insulin secretion by inhibiting the production of inflammatory cytokines and eicosanoids and adipokines from adipose tissue [74]. According to the study, supplementing omega 3 PUFAs improves insulin sensitivity and glucose homeostasis regulation, thereby reducing the risk of developing T2DM [147]. White adipose tissue (WAT) inflammation is increased by high-fat diet models, which also decreases insulin sensitivity and activates Toll-like receptor 4 signaling [148]. A systematic review of the evidence from HFD interventions found that administering HDF for 2 days to 6 weeks to both lean individuals and those with a fat intake of 45–83% as well as overweight or obese individuals increased fatty acid oxidation and maintained impaired insulin sensitivity [149]. In a rat experiment, giving non-glandular Goto-Kakizaki rats HFD increased beta cell dysfunction [150].

**Fig. 2:**
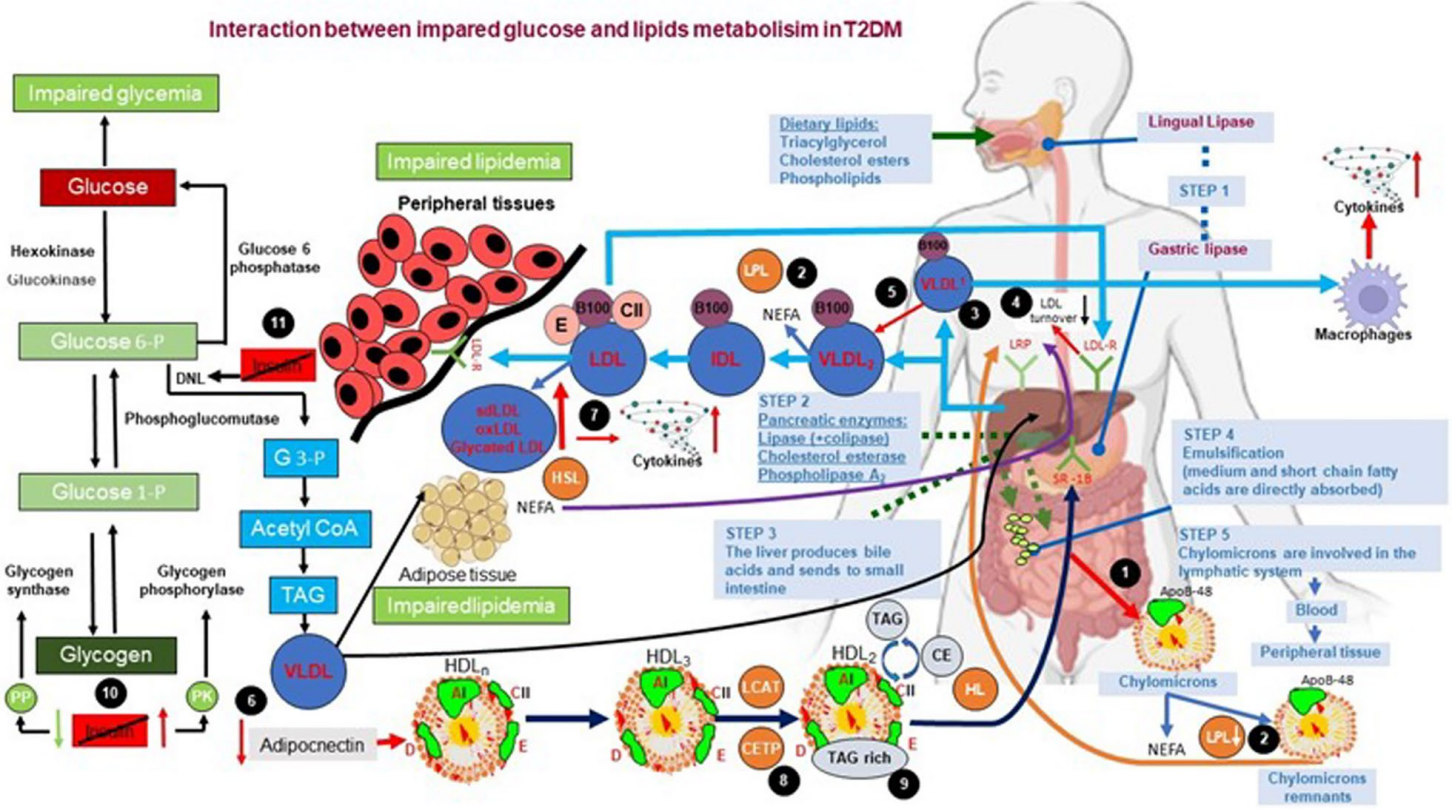
Interaction between impared glucose and lipids metabolism in T2DM. An overview of Interaction between impared glucose and lipids metabolisim inT2DM. (1) increased chylomicron production, (2) reduced catabolism of both chylomicrons and VLDLs (diminished LPL activity), (3)increased VLDL production (mostly VLDL1), (4) reduced LDL turnover (5) increased production of large VLDL (VLDL1) preferentially taken up by macrophages; LDL (qualitative and kinetic abnormalities): (6) low plasma adiponectin favouring the increase in HDL catabolism. (7) increased number of glycated LDLs, small, dense LDLs (TAG-rich) and oxidised LDLs, which are preferentially taken up by macrophages; (8) increased CETP activity (increased transfer of triacylglycerols from TAG-rich lipoproteins to LDLs and HDLs), (9) increased TAG content of HDLs, promoting HL activity and HDL catabolism, (10) İmpaired glucose metabolisim. (11) İmpaired de novo lipid metabolisim (Acetyl CoA and NADPH inhibit pyruvate dehydrogenase as a result of B oxidation. The lactate and alalnin thus formed increase hyperglycemia because of gluconeogenesis (ketone bodies formation increases) in the liver.) *CE*, cholesterol ester, *CETP* cholesteryl ester transfer protein, *HDLn* nascent HDL, *HL* hepatic lipase, *HSL* hormone-sensitive lipase, *LPL* lipoprotein lipase, *SR-B1* scavenger receptor B1, *TAG* triacylglycerol, *PP* protein phosphatase, *PK* protein kinase, NEFA non-esterified fatty acids, *DNL* de novo lipogenesis, *LCAT* Lesitin-kolesterol açil transferaz, *G3P* gliserol 3-fosfat protein kinase

Adipocytokines are signaling proteins that play key roles in the regulation of lipid and glucose metabolism, the neuroendocrine system, and the immune system, as well as energy homeostasis [151]. Adiponectin is also an anti-inflammatory adipokine that regulates the synthesis of FAs, glucose uptake, and fatty acid β-oxidation [152]. The expression of adiponectin, its induction by insulin, and its modulation of β-cell function form the basis of the association between adiponectin and insulin resistance/hyperinsulinemia [153]. According to studies, high TNF-α levels and hypoadiponectinemia both contribute to insulin resistance [154]. The study found that people with T2DM have significantly lower serum adiponectin concentrations, which can be a therapeutic parameter for treating people with T2DM [155]. Serum HDL concentrations and circulating adiponectin levels exhibit a strong positive correlation [156]. The major HDL ApoA-I and the ATP-binding cassette transporter A1 (ABCA1), which has the nuclear receptors liver X receptor and PPARγ, are produced more often in the liver when adiponectin is present. Additionally, the formation of large HDL particles (HDL2) from small HDL particles, which is adiponectin's mechanism for reducing HL activity, increases HDL-C (HDL3). In particular, triglyceride hydrolysis in VLDL particles may be increased by adiponectin via upregulation of LPL, reducing triglyceride transfer to HDL [157]. Adiponectin release is influenced by impaired insulin metabolism, which in turn affects lipid metabolism. The development of low HDL-C concentrations is influenced by insulin resistance through many mechanisms. First off, as IR's influence on CETP, which controls insulin, declines, HDL-C levels rise, possibly because of decreased apo A-I production and secretion from the liver and intestine and increased HL. The formation of TG-rich lipoprotein-derived HDL particles is reduced, and HL formation is increased, because of decreased LPL activity. This is because less TG is hydrolyzed from chylomicrons and VLDL [158]. The pathogenesis of T2DM in relation to glucose and lidipi metabolism may also be influenced by adipokines like chemerin, leptin, fetuin-A, retinal binding protein 4, vaspin apelin, nesfatin-1, and dipeptidyl peptidase-4 [159]. Impaired insulin metabolism in T2DM increases lipogenesis, which disrupts lipid metabolism by decreasing glycogen synthesis and glucose metabolism via the TCA cycle [160]. The effect of the regulation of impaired insulin on glucose and lipid metabolism is presented in Fig. 2.

Lipidomics, or, more generally, metabolomics, is sensitive to various variables, including the host genotype, s gut microbiota, and diet [161]. The immune system and many other processes, including inflammation, incretin secretion, glucose homeostasis, production of short-chain fatty acids (SCFAs), and bile acid metabolism, are regulated by the intestinal microbiota. Intestinal barrier integrity, pancreatic β-cell proliferation, and short-chain fatty acid synthesis, which supports insulin biosynthesis, can all be negatively impacted by the dysbiosis of the gut microbiota, which can disrupt glucose homeostasis and lead to the onset of T2DM [162, 163]. Different microorganisms can produce different products in metabolomics and lipidomics because each has unique properties [164]. The development of insulin resistance and T2DM may be significantly influenced by FAs. FAs' long-term impact on T2DM is not yet been fully understood, though. Although lipidomics is a relatively underutilized tool, it is used more frequently than genomics, transcriptomics, and proteomics to advance our understanding of obesity and T2DM. A subfield of metabolomics called lipidomics may help us better understand how FAs and lipids, particularly insulin resistance and T2DM, contribute to the emergence of health-related complications [165]. Research on T2DM-related gut flora disorders will advance with the integration of gut metabolomics and metagenomics [166].

## Dietary lipids and lipidomics in T2DM via gut-brain axis

Although dietary lipids and lipidomics play an important role in the development of type T2DM, their effect mechanisms have not yet been fully elucidated. However, the gut-brain axis plays a role in glucose homeostasis [167]. The gut-brain axis is a bidirectional communication pathway. Signals from the brain communicate with the gut via the autonomic nervous system and the hypothalamic-pituitary axis to regulate many physiological processes. Signals from the gut to the brain are mediated by vagal and spinal afferent neurons [168]. The gut-brain axis contains numerous components, containing highly specialized cells responsible for transmitting the information. These are the enteroendocrine cells (EEC), central nervous system (CNS), enteric nervous system (ENS), vagus nerve, and gut microbiota. EECs are specialized trans-epithelial cells found throughout the gut [169]. The ENS, the nervous system of the gastrointestinal tract, consists of various types of neurons, including intrinsic primary afferent and motor neurons [170]. It contains 200 to 400 million neurons and enteric glial cells. It also extends throughout the gastrointestinal tract from the esophagus to the anus [169]. Glucose homeostasis is provided via the CNS and ENS in the gut-brain axis. Ghrelin and glucagon-like peptide-1 (GLP-1) have emerged as the key factors that can transmit metabolic information to the brain and stimulate endogenous glucose production and usage [171]. The vagus nerve transmits information related to food intake to the brain to regulate energy and glucose homeostasis [169]. Therefore, it is thought that changes in the gut-brain axis may cause T2DM [170].

Gut microbiota is also considered an important part of the gut-brain axis, so the microbiota-gut-brain axis is emerging as a prominent factor nowadays [169]. Gut microbiota is a collection of more than 100 trillion microorganisms (bacteria, fungi, protozoa, and viruses) and their genomes found in the gastrointestinal tract [172]. Intestinal microorganisms colonize the gastrointestinal tract and contribute to homeostatic balance under healthy physiological conditions [173]. While the host provides a nutrient-rich environment for microorganisms, microorganisms also affect host physiology, immunology, and metabolism [174]. Gut microbiota is the main mediator of the gut-brain axis in the regulation of glucose homeostasis. It contributes to glucose homeostasis by regulating the immune system, inflammatory response, modulation of incretin secretion, production of SCFA, and metabolism of bile acids [171]. Gut microbiota produces several metabolites that directly and indirectly affect the gut-brain axis. GLP-1 is an endocrine factor that may be involved in the control of the gut-brain axis by the gut microbiota [170]. Cani et al. (2006) demonstrated that the modulation of gut microbiota improves glucose metabolism via a GLP-1-dependent mechanism [175]. Gut microbiota can affect serotonin (5-HT) production by EECs, altering ENS vagal afferent activation. Bile acids can also alter the expression of Takeda-G-protein-receptor-5 (TGR5) and affect intestinal peptide release from EECs. SCFAs can alter nutrient receptor expression and intestinal peptide production by EECs or can directly activate vagal afferents. Lipopolysaccharides (LPS), a products of pathogenic microorganisms, can impair gut-brain signaling by preventing the activation of vagal afferents or the ENS [168].

**Fig. 3:**
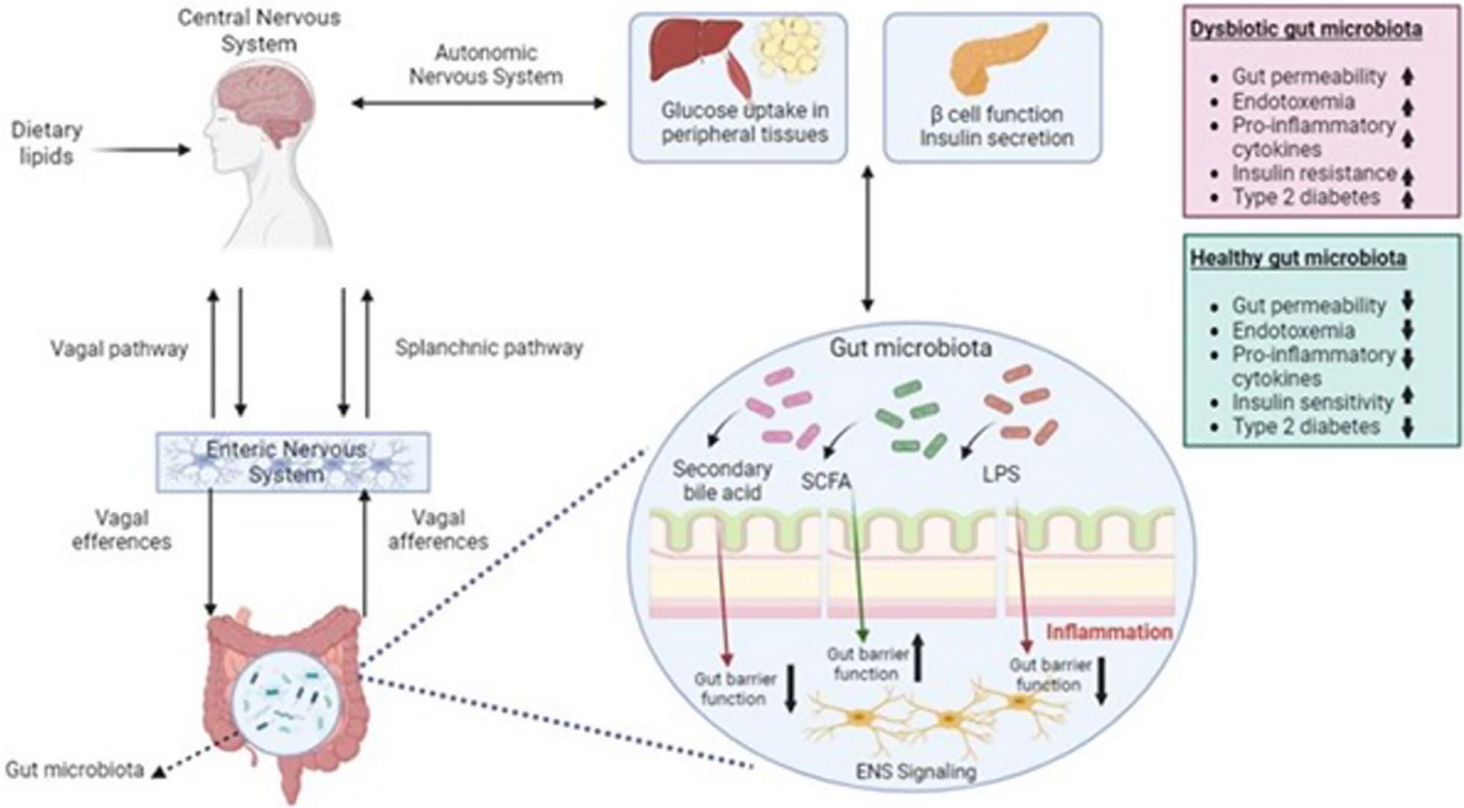
Gut-brain axis pathways related to T2DM. *ENS* enteric nervous system, *SCFA* short-chain fatty acid, *LPS* lipopolysaccharide

Microbiota dysbiosis is the disruption of intestinal homeostasis by altering the composition of the gut microbiota [172]. A direct causal relationship between gut dysbiosis and the development of has not been identified. However, immunomodulatory mechanisms mediated by microbiota-derived lipids have been discovered. The most well-known is the release of LPS and decreased SCFA production, which have pro-inflammatory effects. Other suggested mechanisms include altered bile acid metabolism, altered secretion of incretin hormones such as GLP-1, altered circulating branched-chain amino acids, and impaired adipose tissue, liver, or skeletal muscle functions [163, 176]. This results in increased colonic permeability, colon, liver, and adipose tissue inflammation, impaired insulin secretion, the occurrence of insulin resistance, impaired glucose and lipid metabolism, and the development of T2DM [163]. Microbiota dysbiosis in T2DM is characterized with decreased numbers of SCFA-producing gram-positive bacteria and increased numbers of LPS-producing gram-negative opportunistic pathogens [176]. In a recent study, T2DM patients exhibited a higher *Firmicutes/Bacteroidetes* ratio than healthy individuals. Bacterial diversity in the gut microbiota was reduced in patients with prediabetes or T2DM compared with healthy individuals [163]. In another study, it was observed that T2DM patients had a decreased concentration of *Faecalibacterium prausnitzii*. It has anti-inflammatory properties, promotes the proliferation and growth of epithelial cells and increases the synthesis of tight junction proteins. *Ruminococcus bromii*, which contributes to the production of SCFAs, was also reduced in T2DM patients [177]. A study showed that the colonization of germ-free (GF) mice with healthy gut microbiota caused the restoration of neuronal GLP-1 and ENS signaling pathways in the gut. However, it was indicated that this effect was eliminated by colonization with the diabetic gut microbiota [178]. Another study showed that transplantation of fecal microbiota from lean donors to individuals with metabolic syndrome caused improved insulin sensitivity in the recipients. This suggests that healthy gut microbiota can improve metabolic outcomes [179]. Furthermore, the improvement in glucose homeostasis is associated with GLP-1R signaling, indicating that prebiotic-induced changes in the microbiota restore the gut-brain axis [175]. Promoting the growth of beneficial bacteria using indigestible carbohydrates can improve glucose tolerance [180]. The gut-brain axis pathways related to T2DM was shown in Fig. 3.

## Dietary lipids in T2DM via gut-brain axis

Dietary lipids affect the gut microbiota composition, metabolic-end products, other enzymatic markers, and all microbiota-related diseases [181]. However, the mechanisms of the interplay between dietary lipids and gut microbiota in glucose homeostasis have not been well defined. Many dietary FAs are absorbed in the small intestine; however, some of them directly affect the colonic microbiota composition. It has been shown that gut microbiota can affect glucose and lipid metabolism and may even cause T2DM by disrupting the balance between pro-inflammatory and anti-inflammatory effects in the liver. A diet rich in saturated FAs can adversely affect the microbiota composition and reduce insulin sensitivity [182]. A high-fat diet increases the amount of *Firmicutes* and decreases the amount of *Bacteroidetes*, which may lead to the development of T2DM [183]. Conversely, a diet high in omega 3 FAs and MUFA may promote a favorable alteration of microbiota composition in T2DM patients [87, 184]. Table 2 summarizes the role of dietary lipids on gut microbiota and T2DM.

**Table 2:** Role of dietary lipids in gut microbiota in T2DM

Dietary lipids	Effects on the gut microbiota	Outcomes of glucose homeostasis	References
High fat	Gut microbiota diversity ↓ Gram-negative bacteria ↑ *Bifidobacterium* ↓ *Desulfovibrio* ↑ *Firmicutes/Bacteriodetes* ratio ↑ Intestinal mucus thickness ↓ LPS translocation ↑ Intestinal permeability ↑ Metabolic endotoxemia ↑ Proinflammatory cytokines ↑ Low-grade inflammation ↑	GLP-1 release ↓ Pancreatic β-cell function ↓ Hyperinsulinemia ↑ Hyperglycemia ↑ Glucose intolerance ↑ Insulin resistance ↑ T2DM ↑	[146, 167, 178, 182, 183, 187–189, 197]
Saturated fatty acids	Gut microbiota diversity ↓ *Firmicutes* ↑ *Bacteroidetes* ↓ *Proteobacteria* ↑ *Bifidobacterium* ↓ *Akkermansia muciniphila* ↓ *Lactobacillus* ↓ Intestinal mucus thickness ↓ Intestinal permeability ↑ Butyrate production ↓ Systemic inflammation ↑	Insulin sensitivity ↓ Glucose intolerance ↑ Insulin resistance ↑ T2DM ↑	[179, 186, 198–201]
Omega 3 fatty acids	Gut microbiota diversity ↑ *Enterobacteriaceae* ↓ *Bifidobacterium* ↑ Akkermansia ↑ *Firmicutes* ↓ *Firmicutes/Bacteriodetes* ratio ↓ LPS translocation ↓ Proinflammatory cytokines ↓ Intestinal inflammation ↓ SCFA production ↑ Metabolic endotoxemia ↓	Insulin resistance ↓ T2DM ↓	[85, 179, 203–206, 208, 209]
Omega 6 fatty acids	Gut microbiota diversity ↓ *Firmicutes* ↑ *Actinobacteria* ↑ *Proteobacteria* ↑ *Bacteroidetes* ↓ SCFA production ↓ Secondary bile acid production ↑	Glucose intolerance ↑ T2DM ↑	[199, 210, 211]

### High fat

High-fat diets cause decreased gut microbiota diversity, increased number of gram-negative bacteria, increased LPS translocation, increased intestinal permeability, systemic inflammation, and impaired immune system [184].

High-fat diets cause the loss of beneficial microorganisms and disrupt the symbiotic relationship between the gut microbiota and the host [185]. High-fat diets are associated with lower *Bifidobacterium* species and higher plasma LPS concentration. Likewise, it has been observed that the growth of *Desulfovibrio* bacteria, which are gram-negative, opportunistic pathogens and produce LPS, during high-fat feeding in mice [148]. LPS, also known as endotoxin, is a structural compound in the outer membrane of gram-negative bacteria [103]. LPS initiates low-grade inflammation by activating Toll-Like Receptor 4 (TLR-4), which is expressed in macrophages, hepatocytes, and adipocytes [172]. This mechanism includes several steps. In the first step, LPS binds to lipopolysaccharide-binding proteins (LBP) and interacts with a cluster-of-differentiation 14 (CD14), which is a glycosyl-phosphatidylinositol-anchored protein. In the second step, TLR-4 is activated, triggering a signaling cascade that ends with phosphorylation and activation of focal adhesion kinase (FAK) in enterocytes. In the third step, FAK increases intestinal permeability by regulating IL-1R-associated kinase 4 (IRAK4)-related myeloid differentiation primary response gene 88 (MyD88) activation. In the final step, LPS translocates into the systemic circulation and causes the release of interleukins. IL-6 causes a low-grade inflammation that affects insulin signaling and triggers insulin resistance. This step constitutes the onset of pancreatic β-cell dysfunction [177]. Additionally, LPS stimulates the innate immune signaling cascade by enhancing the expression of inducible nitric oxide synthase (iNOS), which is an effector of the innate immune system and synthesizes nitric oxide. iNOS disrupts insulin receptor substrate-1 (IRS-1) expression. Thus, this can result in hyperinsulinemia, insulin resistance, and T2DM [172].

A healthy intestinal epithelium acts as a barrier to preventing the migration of bacterial-derived factors and prevents LPS from entering the systemic circulation [103]. However, high-fat diets disrupt intestinal barrier function, allowing LPS translocation. This condition, called metabolic endotoxemia, can cause decreased pancreatic β-cell function, insulin resistance, and T2DM [184].

Animal studies have revealed that long-term high-fat feeding is associated with increased circulating LPS [186, 187]. Cani et al. (2007, 2008) reported that endotoxins were approximately 1.5 times higher in lean and obese mice fed a high-fat diet (72% of total energy) than in mice fed a normal diet for four weeks [180, 188]. In previous studies, mice fed a high-fat diet developed hyperglycemia, hyperinsulinemia, glucose intolerance, and insulin resistance, which are associated with decreased intestinal mucus thickness and increased intestinal permeability [180, 189, 190]. In an animal study, mice were fed a low-fat (10% of total energy) or high-fat (60% of total energy) diet for eight weeks. High-fat diets increased proinflammatory cytokines, plasma endotoxin levels, and *Firmicutes/Bacteriodetes* ratio, resulting in intestinal dysbiosis. Additionally, fasting blood glucose and insulin concentrations were higher in high-fat-fed mice than in low-fat-fed mice [191]. Richards et al. (2016) showed that GLP-1 release was reduced in mice fed a high-fat diet (60% of total energy) for two weeks. They also found that the expression of L-cell-specific genes decreased in mice fed a high-fat diet for sixteen weeks. This suggests a disruption in enteroendocrine cell function and gut-brain axis [169].

High-fat diets have been associated with increased LPS levels in humans [192, 193]. Ghanim et al. (2009) showed that a high-fat and high-carbohydrate meal increased the LPS levels and TLR-4 expression [194]. Liang et al. (2013) reported that high LPS levels were negatively associated with skeletal muscle insulin sensitivity in obese individuals with or without T2DM [195]. A study conducted in humans found that endotoxin levels increased in individuals with glucose intolerance and T2DM (respectively, 20% and 125%) [193]. Gomes et al. (2017) reported that LPS levels in patients with diabetes were 66.4% higher than in nondiabetic patients. Additively, it was shown that TLR-4 expression was higher in patients with diabetes compared to nondiabetic patients [196]. It was evidenced that mice lacking CD14, a co-receptor of TLR-4, were more hyperinsulinemia-resistant and insulin resistance induced by a high-fat diet or LPS [188]. Therefore, deletion or mutation of the gene encoding TLR-4 may protect against fatty acid-induced insulin resistance and T2DM [197, 198]. Hulston et al. (2015) reported that a high-fat diet (65% of total energy) impaired insulin sensitivity in healthy and non-obese individuals. Besides, probiotic supplementation (*Lactobacillus casei*) for four weeks maintained normal insulin sensitivity [199]. Animal and human studies indicate that high-fat diets may cause the development of T2DM by affecting the gut-brain axis and gut microbiota.

### Saturated fatty acids

SFAs have been associated with increased non-commensal bacteria (*Firmicutes* and *Proteobacteria*), intestinal barrier dysfunction, decreased gut microbiota diversity, thinning of the mucus layer, decreased butyrate-producing bacteria, chronic inflammation, and development of T2DM [181]. addition SFAs cause ER stress. ER stress-associated systemic inflammation also induces disruptions in insulin signaling pathways. Systemic inflammation is considered a precursor to the development and progression of insulin resistance [172].

Animal studies have found that a high-fat diet increased *Firmicutes* and decreases *Bacteroidetes* in the gut microbiota. This is associated with insulin resistance resulting from intestinal inflammation [188, 200, 201]. An animal study showed that a saturated fat diet increased insulin resistance, intestinal permeability, and mesenteric fat inflammation [202]. Caesar et al. (2015) compared rats fed a high-fat diet rich in SFAs as lard to an isocaloric high-fat diet rich in omega 3 FAs as fish oil. It was found that *Akkermansia muciniphila*, *Lactobacillus*, and *Bifidobacterium*, which are beneficial bacteria, were less in the microbiota of the mice fed a high-fat lard diet. Moreover, a high-fat lard diet activates TLR-4 signaling, reducing insulin sensitivity and increasing inflammation in white adipose tissue [203]. In an animal study comparing lard-based and palm oil-based diets, lard-based diets were associated with impaired glucose tolerance. Lard-based diets also alter gut microbiota composition and function, acting on lipid metabolism [204]. Although there is a limited number of human intervention studies on SFAs, data from animal studies suggest that SFAs causes intestinal dysbiosis, leading to insulin resistance and T2DM.

### Omega 3 fatty acids

Omega 3 FAs can prevent insulin resistance and T2DM development by increasing the diversity of the gut microbiota, reducing LPS and proinflammatory cytokines, and increasing SCFA production. Omega 3 FAs may exert beneficial effects on the gut microbiota by reducing the proliferation of *Enterobacteriaceae*, increasing the proliferation of *Bifidobacterium*, and subsequently inhibiting the inflammatory response associated with metabolic endotoxemia [205]. Besides, it was reported that a high intake of omega 3 FAs was associated with an increased translocation of commensal bacteria (*Bifidobacterium* and *Akkermansia*) and a decreased *Firmicutes/Bacteroidetes* ratio [181]. Omega 3 FAs inhibit LPS-induced pro-inflammatory cytokine production in human blood monocytes and attenuate intestinal inflammation [206]. However, omega 3 fatty acid supplementation has not yet been demonstrated to affect insulin metabolism and gut microbiota [87].

Patterson et al. (2014) showed that a flaxseed/fish oil diet for sixteen weeks significantly increased the intestinal population of *Bifidobacterium* in mice [207]. Administration of omega 3 FAs to *Salmonella*-infected mice increased the amount of SCFAs, altered the gut microbiota, and supported host resistance to pathogens [208]. In a prospective study, thirty-five patients with T2DM were randomly assigned to a standard T2DM diet group and a standard T2DM diets enriched with 100 g of sardines group. There was no significant difference between the glycemic controls of the patients in either groups. Plasma insulin and insulin resistance (HOMA-IR) decreased from baseline in both groups. *Firmicutes* decreased in both groups after six months of intervention. Patients fed a standard T2DM diet enriched with sardines had a decreased *Firmicutes/Bacteroidetes* ratio [87]. The dietary intake of fish oil has increased the diversity of intestinal flora more than sunflower oil [209]. A diet high in omega 3 FAs has been observed to increase the number of several SCFA-producing bacteria in the human gut microbiota, including *Blautia*, *Bacteroides*, *Roseburia*, and *Coprococcus* [210]. An animal study showed that alpha-linolenic acid-rich flaxseed oil significantly reduced fasting blood glucose, HbA1c, LPS, IL-1β, TNF-α, and IL-6 levels. Additionally, flaxseed oil intervention increased acetate, propionate and butyrate levels. Flaxseed oil eased T2DM by suppressing inflammation and regulating the gut microbiota. *Firmicutes* and *Firmicutes/Bacteroidetes* ratios decreased, while *Bacteroidetes* increased [211]. All these studies suggest that omega 3 FAs reduces the risk of T2DM development by increasing SCFA production. However, data from both animal models and human interventions are not enough and uncertain.

### Omega 6 fatty acids

Animal studies have shown that high-fat diets containing large amounts of omega 6 FAs increase *Firmicutes*, *Actinobacteria*, and *Proteobacteria* and decrease *Bacteroidetes* [201, 212]. Thus, omega 6 FAs may cause T2DM, raising intestinal dysbiosis. Miao et al. (2020) showed that both dietary omega 6 FAs and circulating omega 6 FAs (gamma-linolenic acid) could increase the risk of T2DM through a mechanism that alters the diversity and composition of the gut microbiota. Additionally, gamma-linolenic acid was inversely related to butyrate-producing bacteria in this study. Omega 6 FAs can increase the secretion of bile acid, which may act as an important signaling molecule linking omega 6 FAs and the gut microbiota. Bile acids can be metabolized in the gut to deoxycholic acid, which can disrupt hepatic ER stress. Therefore, it can disrupt glucose homeostasis and cause T2DM [213]. Although omega 6 FAs may play a role in the development of T2DM by the microbiota-gut-brain axis, more animal and human studies are needed in this field.

## Lipidomics in T2DM via gut-brain axis

Lipidomics plays a major role in glucose homeostasis and the development of T2DM. It has been shown that the gut microbiota composition is altered in individuals with T2DM. However, the relationship between gut microbiota and lipidomics needs to be clarified in T2DM [214]. Animal studies suggest that the gut microbiota may modulate the lipidomics of the host. Homeostasis between lipidomics and gut microbiota is also known to affect the metabolic state of the host [204]. Furthermore, gut microbiota can synthesize lipids and their metabolites that may impact human health. Changes in gut microbiota and host lipidome appear to be associated with the development of T2DM [215]. However, there are few studies on lipidomics and its relationship to the gut-brain axis in T2DM [216].

### Sphingolipids

Sphingolipids are bioactive lipids that regulate cellular processes such as cell differentiation, proliferation, apoptosis, and inflammation. Sphingolipids can be obtained either by diet or de novo synthesis [215]. However, it has been reported recently that the commensal gut microbiota (*Bacteroides*, *Prevotella*, and *Porphyromonas*) also produces sphingolipids. Ceramide phosphoinositol and deoxy-sphingolipids are synthesized by gut microbiota. These sphingolipids can exacerbate intestinal inflammation and regulate the amount of ceramide in animals. Sphingolipids may be an early marker of impaired glucose metabolism, but there are limited human studies with conflicting results [217].

Ceramides are precursors to sphingolipids [218]. Ceramides may be associated with insulin resistance because they may interfere with insulin signaling. It has been reported that ceramide levels are increased in serum, liver, and skeletal muscle in T2DM patients. Ceramides can trigger adipose tissue inflammation and diabetic pathology, activating pro-inflammatory cytokines [219]. In another study, ceramides were found to be positively associated with HOMA-IR in patients with T2DM [220]. Holland et al. (2011) showed that ceramides mediated saturated FAs-induced insulin resistance by TLR-4 signaling in skeletal muscle [218]. In 435 American-Indian participants in the Strong Heart Family Study, higher levels of ceramides, including stearic acid, arachidic acid, and behenic acid, were associated with T2DM [221]. In another study conducted in China, ceramides (18:1/18:1, 18:1/20:0, 18:1/20:1, 18:1/22:1), saturated sphingomyelins (C34:0, C36:0, C38:0, C40:0), unsaturated sphingomyelins (C34:1, C36:1, C42:3), hydroxyl-sphingomyelins (C34:1, C38:3) and hexosylceramide (d18:1/20: 1) was associated with T2DM. This study demonstrated that high levels of ceramides and sphingomyelins accompany pancreatic β-cell dysfunction [23].

Many lipidomics other than ceramides has been defined in T2DM patients. Thirty-five newly diagnosed T2DM patients had higher levels of sphingomyelins (d18:1/18:0, d18:1/18:1), ceramides (d18:1/18:0, d18:1/16:0, d18:1/20:0, d18:1/24:1), lysophosphatidylcholines (15:0, 16:0, 17:0), phosphatidylcholines (19:0/19:0), lysophosphatidylethanolamines (18:0), and phosphatidylethanolamines (16:0/22:6, 18:0/22:6) than the control group. Conversely, phosphatidylethanolamines (17:0/17:0) and phosphatidylcholines (18:1/18:0) were lower in T2DM patients. Levels of serum sphingomyelins (d18:1/18:0, d18:1/18:1), lysophosphatidylcholine (16:0), and lysophosphatidylethanolamines (18:0) decreased after administration of GLP-1 analog [222]. Floegel et al. (2013) associated diacyl-phosphatidylcholines C32:1, C36:1, C38:3, and C40:5 levels with an increased risk of T2DM. On the contrary, sphingomyelin C16:1, acyl-alkyl-phosphatidylcholines C34:3, C40:6, C42:5, C44:4, C44:5 and lysophosphatidylcholine C18:2 were associated with a reduced risk of T2DM [223]. Lysophosphatidylcholine, phosphatidylcholine, phosphatidylethanolamine, and diacylglycerol have been associated with an increased risk of T2DM. Unlike, sphingomyelins have been related to a reduced risk of T2DM [26]. In the Metabolic Syndrome in Men (METSIM) cohort consisting of 10.197 men in total, it was found that levels of triacylglycerol and di-acyl-phospholipids were higher in T2DM patients [224]. Lysophosphatidylcholine was determined to stimulate glucose uptake in 3T3-L1 adipocytes in a dose- and time-dependent manner [225]. Prada et al. (2020) informed a negative correlation between lysophosphatidylcholine and T2DM risk in females. There was a positive correlation between lysophosphatidylcholine and T2DM risk in males. In addition, cholesteryl esters, monoacylglycerols, and diacylglycerols showed an inverse association with T2DM. A positive correlation was found between FFA and T2DM risk [226].

Although sphingolipids seem to be related to glucose metabolism, the effect of the gut-brain axis on this relationship is not yet known. Liu et al. (2019) presented that gestational diabetes was strongly associated with a specific gut microbiota composition and lipidomics. This study showed that *Faecalibacterium* and *Prevotella* species were related to lysophosphatidylethanolamine and phosphatidylglycerol [214]. Further and larger studies are required to investigate the effect of the gut-brain axis on lipidomics and glucose metabolism.

### Bile acids and derivatives

Primary bile acids are synthesized from cholesterol in hepatocytes [168]. Primary bile acids are conjugated with glycine or taurine to form bile salts. About 95% of bile acids are reabsorbed in the ileum into the hepatic portal vein and then into the liver sinusoids. About 400–600 mg of bile salts reach the colon. They are converted to secondary bile acids such as deoxycholic acid, lithocholic acid, and ursodeoxycholic acid by the colonic microbiome [176]. However, secondary bile acids are produced or biotransformed by the gut microbiota. Conjugated primary bile acids are deconjugated by *Bacteroides*, *Clostridium*, *Lactobacillus*, and *Bifidobacterium* species. The gut microbiota plays an important role in bile acid metabolism. In addition, both primary and secondary bile acids affect host metabolism [215]. Bile acids regulate glucose homeostasis, lipid metabolism, and gut microbiota [227].

It has been suggested that secondary bile acids may cause T2DM through the gut microbiota. Taurine-conjugated bile acids have been associated with insulin resistance in nondiabetic individuals. They have been significantly higher in patients with T2DM than those without T2DM [228, 229]. In a study, insulin resistance was associated with increased levels of secondary bile acids [230]. In addition, deoxycholic acid, a secondary bile acid, was positively correlated with *Firmicutes* levels [231]. In particular, a high-fat diet alters the gut microbiota, increasing intestinal, brain, and blood levels of taurine-conjugated bile acids such as taurochenodeoxycholic acid, a Farnesoid X receptor (FXR) agonist. Increased taurochenodeoxycholic acid may cause insulin resistance by increasing TCDCA-FXR signaling [232]. There is evidence that microbiota dysbiosis can cause T2DM by increasing secondary bile acids; however further animal and human studies are required.

### Endocannabinoids

The endocannabinoid system consists of lipid-derived endogenous ligands, enzymes involved in their synthesis and degradation, and cannabinoid receptors [215].

Gut microbiota has close linked to the endocannabinoid system. It has been suggested that the gut microbiota and endocannabinoids may communicate with signaling pathways involving the gut-brain axis for homeostasis of energy, lipid, and glucose metabolism. N-acyl phosphatidylethanolamine-specific phospholipase D regulates lipid absorption and metabolism. Its disruption causes insulin resistance, glucose tolerance, and intestinal dysbiosis [215]. Muccioli et al. (2010) demonstrated a relationship between the gut microbiota and the endocannabinoid system that modulates host adipogenesis [233]. Everard et al. (2013) showed that *Akkermansia muciniphila* intervention in obese mice increased intestinal levels of 2-oleoylglycerol, 2-arachidonoylglycerol, and 2-palmitoyl-glycerol [189]. More recently, it was reported that the endocannabinoid-like molecule N-acyl-3-hydroxypalmitoyl-glycine was synthesized by *Bacteroides* species [234]. These studies suggest that the gut microbiota generates lipidomics that influences signaling pathways of host metabolism. The mechanisms related to the gut microbiota that mediate the increase in intestinal permeability have not been fully elucidated. However, overactivation of the endocannabinoid system may play an important role in intestinal permeability [235]. In addition, endocannabinoids can affect the composition of the gut microbiota. Deletion of the endocannabinoid-synthesizing enzyme in adipose tissue may cause obesity, glucose intolerance, altered lipid metabolism, and adipose tissue inflammation [236].

### Specialized pro-resolving mediators

Specialized pro-resolving mediators (SPMs) are synthesized from AA, eicosapentaenoic acid, docosahexaenoic acid, and docosapentaenoic acid during inflammation. These endogenous lipids promote the clearance of pathogenic bacteria and macrophages associated with microbiota dysbiosis. They also enhance tissue regeneration by increasing the secretion of anti-inflammatory mediators and inhibiting proinflammatory cytokines. These lipid mediators play a crucial role in eliminating inflammation, maintaining intestinal integrity, and preventing T2DM [181].

### Short chain fatty acids

SCFA, which are organic FAs containing 2–6 carbon atoms, are synthesized in the host's colon by the microbiota following fermentation of indigestible dietary fibers, proteins, and glycoproteins. Acetate, propionate, and butyrate account for 95% of SCFAs present in the colon [170]. SCFA-producing commensal bacteria include *Lachnospira*, *Akkermansia*, *Bifidobacterium*, *Lactobacillus*, *Ruminococcus*, *Roseburia*, *Clostridium*, *Faecalibacterium*, and *Dorea*. SCFAs, which are used as substrates for energy production, lipogenesis, gluconeogenesis, and cholesterol synthesis, affect host metabolism. SCFAs act as signaling molecules by stimulating G protein-coupled receptors GPR43/FFAR2 and GPR41/FFAR3. GPR43 increases GLP-1 expression by affecting L cells. Acetate regulates glucose and lipid metabolism via GPR43 [215]. Acetate and propionate exert anti-inflammatory effects by FFAR2. This receptor signaling results in the inhibition of NF-kB nuclear translocation and decreased expression of proinflammatory cytokines such as TNF-α, IL-1, and IL-6. Preventing inflammation can also reduce the risk of T2DM [177].

Microbiota dysbiosis can cause T2DM development by decreasing SCFA concentrations [237]. The composition of the gut microbiota and diet affect the type and amount of SCFA. High-fat diets and some FAs can result in decreased SCFA production and increased harmful secondary metabolite production. Increased intestinal permeability can cause low-grade inflammation and metabolic endotoxemia. Metabolic endotoxemia can also cause insulin resistance, β-cell dysfunction, hyperglycemia, and T2DM [238]. High-level endotoxemia has been found to increase TNF-α and IL-6 concentrations and insulin resistance [239]. Recent evidence suggests that dietary lipids and lipidomics can cause T2DM by reducing SCFA production. However, the effect of SCFAs on the pathophysiology of T2DM has generally been observed in animal studies, but these results should be confirmed in larger, long-term studies with clinical endpoints.

## Nutritional strategies modulating gut microbiota through lipidomics for T2DM

**Fig. 4:**
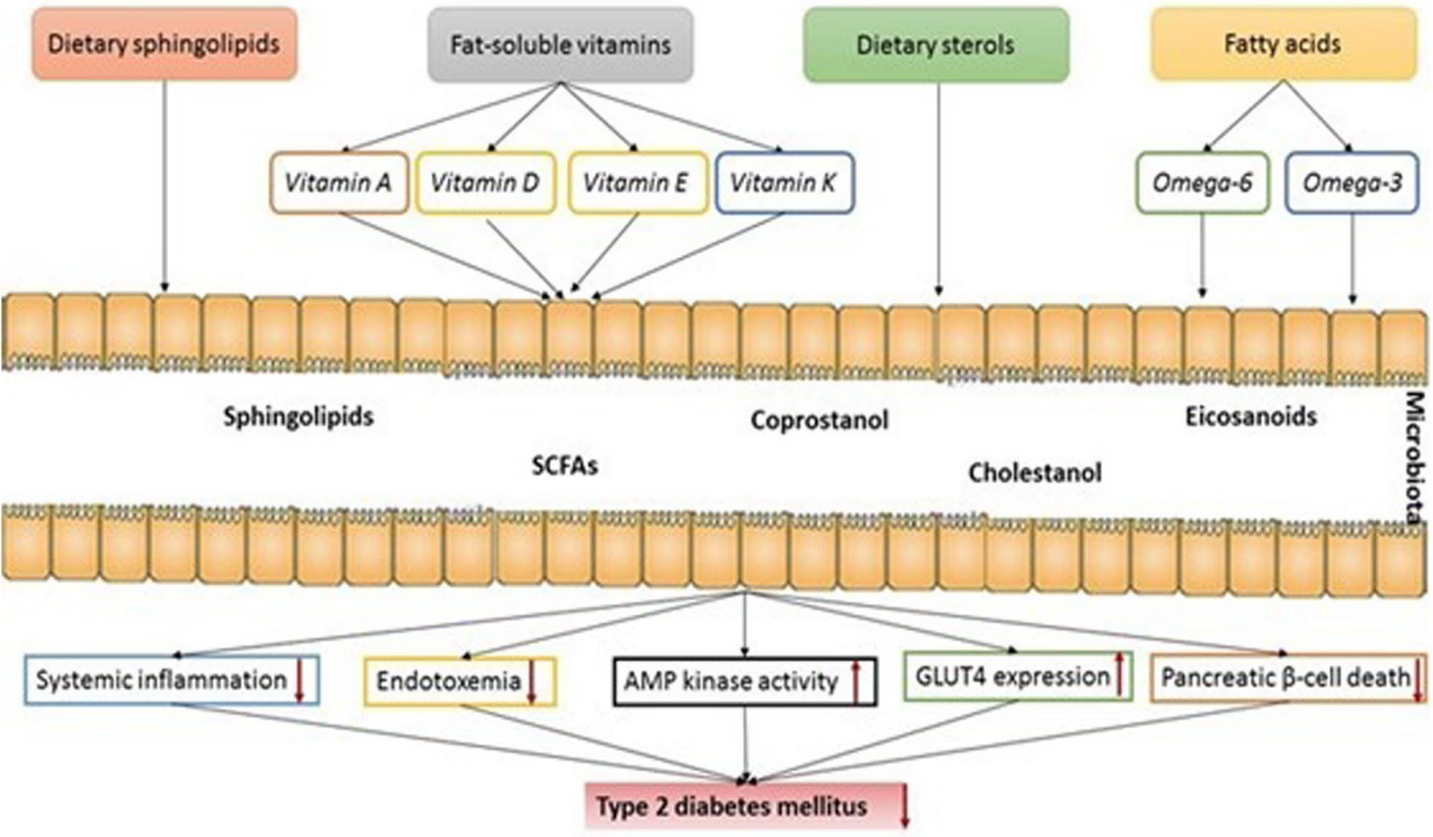
Some nutritional strategies in T2DM considering lipidomics and gut microbiota interaction

To promote health, it is now essential to understand how nutrients impact metabolic control [240]. It is well known that a wide range of exogenous and intrinsic factors affect the intestinal microbiota. The human gut microbiota can be shaped and modified by a number of factors, but diet is one of the most potent [241]. Food is the primary source of energy for microorganisms in the gut, and changes in the host's diet can result in rapid changes in the microbiota's composition [241, 242]. Up to 57% of changes in the gut microbiota may be attributed to dietary factors, but host genes are only thought to be responsible for 12% or less of those changes [243]. To combat noncommunicable diseases like T2DM, including understanding how diet and dietary nutrient intake affect the gut microbiome, is crucial [244]. This makes it crucial to identify the dietary components that influence the gut microbiome, where dietary lipids play a significant role [181, 244]. Roles and mechanisms of lipidomics in T2DM were discussed in the sections before this one. It is possible that T2DM can be prevented and treated by modulating lipidomics, according to this. A key role in the modulation of lipidomics is played by nutritional status and dietary nutrients. It is stated that especially dietary lipid intake may have anti-diabetic effects with the changes it has created on the composition of the intestinal microbiota through metabolic end products [211, 245–247]. The possible roles that some nutritional strategies may play in T2DM through lipidomics and gut microbiota were shown in Fig. 4.

## Fatty acids

### Omega 3 fatty acids

High consumption of fish and seafood is associated with a significantly lower risk of T2DM [248, 249]. It is reported that high omega 3 FAs content may be effective in this situation through different mechanisms such as anti-inflammatory and antioxidant [249–251]. Another potential mechanism is that it lowers the risk of developing T2DM by influencing the intestinal microbiota through lipidomics, which is a substance produced in the intestine as a result of the metabolism of omega 3 FAs [248, 252].

It is known that the intestinal microbiota can be modulated through SCFAs, and in this way, the microbial diversity of the intestine can be changed [253]. SCFAs increase insulin signaling and insulin sensitivity with their curative effects on systemic inflammation and endotoxemia by decreasing intestinal permeability [248]. Additionally, SCFA enhances glucose uptake in skeletal muscle and adipose tissue by boosting GLUT4 expression via AMP Kinase (AMPK) activity [254]. Bacteria that produce butyric acid contribute significantly to maintaining health by converting unfermented dietary fibers into SCFAs such as butyrate [248]. It has been reported that supplementation of omega 3 FAs helps modulate the intestinal microbiota by increasing the level of SCFAs in the intestine [252]. In addition, a diet rich in omega 3 FAs is noted to significantly increase butyrate-producing bacteria, including *Blautia*, *Bacteroides*, *Roseburia,* and *Coprococcus* [248, 252].

Another of the main mechanisms of the protective function of omega 3 FAs is their influence on the lipidome, including eicosanoids and membrane lipids [255]. Yan et al. (2020) found that omega 3 fatty acid supplementation in healthy individuals added glycerophospholipidome levels [255]. In a study carried out with non-obese T2DM rats and Wistar rats, it was detected that dysbiosis occurred and higher glycerophospholipids were present in non-obese T2DM rats when compared with Wistar rats. Another outcome of the study was that omega 3 supplementation significantly affected plasma glycerophospholipids [256]. Considering their effects on the lipidome, these data suggest that omega 3 FAs may influence the intestinal microbiota and subsequently T2DM. In research, the effects of supplements containing varying doses of EPA + DHA and dietary sources of omega 3 such as fish and flaxseed oil on intestinal microbiota were investigated. The research outcomes are influenced by sample size, methodological variations, the study group, and the dose and amount of the supplement or nutritional source utilized.

### Omega 6 fatty acids

Vegetable oils, nuts, and seeds are the main dietary sources of omega 6 PUFAs. The essential omega 6 FAs in the diet are LA and AA. LA makes up the majority (about 90%) of dietary PUFAs, while AA has a lower intake [257]. In the body, the majority of LA is transformed into AA [258]. For several enzymes, AA serves as a substrate [181]. Different biosynthetic pathways (i.e., cyclooxygenases (COX), lipoxygenases (LOX), or cytochrome P450) of these enzymes are used to produce various eicosanoids, including various prostaglandins, thromboxanes, and leukotrienes [259]. According to recent research, the majority of immune cells and intestinal epithelial cells synthesize eicosanoids [181, 260]. Additionally, it is claimed that by metabolizing AA, intestinal bacteria can produce eicosanoid metabolites [181]. Particularly, it has been reported that proteobacterial lipoxygenases can generate a variety of prostaglandins in the intestine by fermenting other bacterial byproducts, primarily SCFAs [181, 261].

Cyclooxygenase pathway metabolites of AA, especially prostaglandins, increase the risk of T2DM by causing pancreatic β-cell destruction and β-cell dysfunction [262]. In line with such data, dietary omega 6 FAs may be considered to role in the development of T2DM through enzyme systems and eicosanoid metabolites produced as a result of their metabolism by intestinal bacteria.

### Dietary sterols

Sterols are lipophilic substances that can be divided into synthetic sterols, phytosterols (derived from plants), zoosterols (derived from animals), and mycosterols (derived from fungi) [263]. About 30–60% of dietary cholesterol is absorbed, while the rate of absorption for plant sterols is much lower (2–3%) [264]. This means that unabsorbed sterols can reach the colon and be further processed by the gut microbiota [181, 264]. Among the common phytosterols, β-sitosterol, stigmasterol, campesterol, and brassicasterol can be found in both plant cells and cytoplasmic membranes of many fruits, vegetables, nuts, cereals, and vegetable oils [263]. Western nations consume about 250 mg of phytosterols per day on average [265].

Metabolites produced as a result of transformation by the gut microbiota of unabsorbed sterols, including cholesterol and plant sterols, have significant effects on the host's health, including in T2DM, through modulation of the gut microbiota [264]. Coprostanol, coprostanone and cholestanol are cholesterol metabolites formed in the gut by the gut microbiota [264, 266]. Coprostanol is the most abundant animal sterol in feces, followed by coprostanone and cholesterol, which can be converted to lesser cholestanol [264].

Plant sterol supplementation was found to increase the production of SCFAs and decrease the production of coprostanol and ethylcoprostanol in a study where the researchers evaluated the effects of plant sterols on the intestinal microbiota (acetate and butyrate). Additionally, it was reported that this supplement could improve the gut microbial profile by raising the percentage of some genera from the phylum Firmicutes [267]. In another study, in which half of 40 postmenopausal women were given a 2 g/day plant sterol-enriched beverage and the remaining half a placebo for six weeks, it was found that the coprostanol levels were lower in the feces of the plant sterol group, and it was concluded that plant sterols could be used to modulate the gut microbiota [268]. In addition, Weststrate et al. discovered that plant sterol-enriched margarine (8.6 g/day) decreased the amount of cholesterol that was converted to coprostanol during metabolism [269]. In line with the data obtained from these studies, it can be concluded that while plant sterols decrease the production of some lipidomics such as coprostanol, they increase the production of SCFAs. As mentioned in earlier sections, SCFAs lower the risk of developing T2DM via a variety of mechanisms, including modulation of the gut microbiome. Furthermore, the gut microbiota and glucose homeostasis are known to be influenced by cholesterol metabolites like coprostanol [268, 270–273]. These results in the literature suggest that some sterols (especially plant sterols) may improve glucose homeostasis and reduce the risk of T2DM by the modulating gut microbiota through lipidomics.

### Fat-soluble vitamins

#### Vitamin A

Vitamin A (retinol) and its enzymatic oxidation product, retinoic acid, play a role in gut health through interactions with the gut microbiome [181, 274, 275]. It has been reported that significant changes in the gut microbiome profile in acute vitamin A deficiency and the abundance of Bacteroides vulgatus increase rapidly [276]. Lv et al. (2016) concluded in their study that children with vitamin A deficiency have less microbial diversity in their gut microbiota and are more likely to experience resistant diarrhea. Another outcome of the study was the detection of less butyrate-producing Clostridia bacteria in the stools of children with vitamin A deficiency [277]. Moreover, it is stated that bacterial diversity may decrease with the decrease in butyrate-producing bacteria and the increase in opportunistic pathogens in case of vitamin A deficiency [277]. It may be considered due to the aforesaid roles that adequate dietary vitamin A intake may reduce the risk of T2DM by increasing butyrate production, which is attributed to lipidomic. To draw firm conclusions, however, is challenging due to the paucity of studies on this topic in the literature.

#### Vitamin D

Vitamin D3 (cholecalciferol), belonging to the secosteroid family, has received increasing attention in recent years for its potential pleiotropic effects on lipid metabolism [278]. Vitamin D, which is known to regulate calcium and phosphate metabolism, also has important effects on blood glucose homeostasis and gut microbiota [279, 280]. Thomas et al. (2020) found out that strong correlations between the vitamin D metabolites 1,25-dihydroxyvitamin D (1,25(OH)_2_D), and 24,25-dihydroxyvitamin D (24,25(OH)_2_D) and intestinal microbial diversity. It has also been reported in the study that the increase in vitamin D metabolites was positively correlated with the increase in butyrate-producing bacteria, including Firmicutes [281]. Oral vitamin D3 supplementation increases the levels of Bacteroides and Parabacteroides in the intestine [282]. In a study, it was determined that vitamin D supplementation increased the levels of probiotic taxa Akkermansia and Bifidobacterium, which are known to have health-improving effects in the gut microbiome, and the ratio of Bacteroidetes/Firmicutes [283]. Haro et al. (2015) also revealed that an increase in Parabacteroides levels in the gut microbiome reduces the risk of T2DM [284]. Moreover, it has been reported that the Firmicutes/Bacteroidetes ratio is higher in T2DM individuals than in healthy individuals [285]. According to these data, it can be considered that vitamin D may reduce the risk of T2DM by affecting the intestinal microbiome through its metabolites.

#### Vitamin E

Vitamin E is a nutrient that can act as an antioxidant and is present in a number of foods, such as wheat germ oil, extra virgin olive oil, hazelnuts, peanuts, fish, oysters, eggs, and butter [244]. There are eight types of vitamin E: α-, β-, γ-, and δ-tocopherol; and α-, β-, y- and δ-tocotrienol. A common form of vitamin E, α-tocopherol, is the most biologically active form in humans [286]. Natural antioxidants are said to be able to regulate the gut microbiome by scavenging too many free radicals and enhancing the immune response [287, 288].

According to a study, mice that consumed high levels of vitamin E had a lower Firmicutes to Bacteroidetes ratio than mice in the control group and mice that consumed low levels of vitamin E [286]. In another study, vitamin E intake was linked to a relative decrease in Proteobacteria [289]. Contrarily, Tang et al. (2016) discovered that supplementing with vitamin E along with iron increased the relative abundance of the butyrate-producing genus Roseburia (phylum Firmicutes) [290]. Yang et al. (2021) came to the conclusion that δTE-13′-carboxychromanol, a metabolite of vitamin E δ-tocotrienol (δTE), prevents Roseburia depletion, which is known to be reduced in people with inflammatory bowel diseases [291]. In an in vitro study in which vitamin E was added to the rumen fluid, acetate and propionate concentrations were increased, but butyrate levels decreased in the rumen fluid [292]. Another in vitro study found that adding vitamin E to the diet increased production of total SCFA, propionate, and tended to increase production of acetate and butyrate [293]. These findings lead to the conclusion that vitamin E plays a role in T2DM by affecting the abundance of some bacteria and indirectly the production of SCFAs.

#### Vitamin K

There are two molecular variations of vitamin K found in nature: vitamin K1 (phylloquinone) and vitamin K2 (menaquinone). The main dietary source of vitamin K is phylloquinone, which is primarily present in green leafy vegetables [294]. Meat, eggs, curd, cheese, fermented soybeans, and dairy products are the main dietary sources of menaquinone. As well as its dietary sources, many bacteria in the human gut also synthesize menaquinone [294, 295]. It has been suggested that vitamin K supplementation may lower the risk of T2DM due to its benefits for improving insulin sensitivity, glucose tolerance, and preventing insulin resistance [294, 296].

Vitamin K plays a role in the proliferation of Bacteroides melaninogenicus that contain sphingolipids in addition to sphingolipid metabolism [297]. It was found that Bacteroides melaninogenicus grown on vitamin K-deficient medium had lower synthase activity than bacteria grown on vitamin K-supplemented medium [297, 298]. It is stated that inhibition of sphingolipid metabolism (decreased sphingolipid metabolism) impairs pancreatic β-cell function and may contribute to the risk of T2DM [299]. On the contrary, there are studies reporting that sphingolipids may contribute to the risk of T2DM by disrupting the insulin signaling pathway and contributing to mitochondrial dysfunction [300, 301].

Obese people with insulin resistance have different gut microbiota compositions, particularly a rise in the Firmicutes/Bacteroidetes ratio [302]. Ellis et al. (2021) found that dietary vitamin K levels affect the gut microbial community composition [303]. Menaquinones, in particular, play a crucial role in gut microbiota homeostasis by encouraging the growth of symbiotic bacteria [181]. Based on the findings of these studies, it is hypothesized that vitamin K may contribute to prevent of T2DM through its effects on the microbiome and sphingolipid metabolism, a lipidomic process. To be certain about the existence and direction of the relationship, new studies are necessary.

#### Dietary sphingolipids

Sphingolipids are lipids that, along with cholesterol, phospholipids, and proteins, play a role in the structure of the cell membrane. There are more than 4000 different subtypes of sphingolipids [304]. Sphingolipids are present in foods in various amounts, ranging from a few micromoles per kilogram in rich sources like dairy products, eggs, and soybeans to a few millimoles per kilogram in fruits. Furthermore, it is known that foods such as beef, pork, chicken, turkey and fish have different levels of sphingolipid content. Sphingolipid consumption per person in the United States is estimated to be between 150 and 180 mmol (∼115–140 g) annually, or between 0.3 and 0.4 g/day, based on the scant information currently available [305]. Western diets contain 200–400 mg/day of sphingolipids and the largest contribution comes from animal sources [305]. The amount of sphingolipids consumed daily from plant sources is thought to be around 50 mg in western countries, but for vegetarians, that amount can be much higher [304]. Milk contains a variety of different types of lipids, such as triglycerides, diglycerides, glycerophospholipids, sphingolipids, NEFA, cholesterols, and glycolipids. Sphingomyelins are the most prevalent of the four types of sphingolipids found in milk, which also includes gangliosides, cerebrosides, and ceramides [306]. Although dietary sphingolipids are not essential for the continuation of life, they play a key role in health, especially their effects on the composition of the gut microbiome [304].

Sphingolipid production by bacteria exists in addition to dietary consumption. Prokaryotic sphingolipid production was found to occur first in members of the Bacteroidetes phylum (e.g. *Bacteroides, Prevotella, Porphyromonas, Sphingobacterium*) and later in some Proteobacteria (eg Sphingomonas, Bdellovibrio, Acetobacter) [304]. The abundance of the Bacteroidetes phylum, which comprises 30–40% of the human gut microbiome, further increases the potential effects of bacterial sphingolipid production on the host [306]. Nutritional status can affect bacterial sphingolipid production by altering the abundance of these bacteria in the gut microbiome [248, 304, 307].

Sphingolipids, which are known to affect the intestinal microbiome, are reported to play a role in the development of insulin resistance and T2DM [300, 301, 304]. These findings suggest that diet may influence T2DM risk by influencing lipidomic sphingolipid intake and indirectly influencing bacterial production.

## Conclusion and future directions

In conclusion, dietary lipids and lipidomics seem to play a major role in glucose homeostasis, the development, and the progression of T2DM. Actually, it has been known for a long time that some dietary and plasma lipids like SFAs do not have positive effects on the development and prognosis of T2DMs, while PUFA and MUFA may have positive effects when intake in balance. The new subject we learned is the effects and mechanisms of action of metabolomics, which we call lipidomics on host health and the gut microbiome. Recent literature has noted that gut microbiota may modulate the lipid profiles and lipidomics in T2DM, however, the gut microbiota is altered in individuals with T2DM. Moreover, lipids and lipidomics may influence the gut-brain axis with certain mechanisms, hence contributing to the pathogenesis of T2DM. Furthermore, some studies in the literature suggest that diet may influence T2DM risk by influencing lipid metabolism and lipidomics and directly or indirectly influencing microbiota composition and forming metabolites because of microbiota modulation. In line with the data obtained from the literature, increasing omega-3 fatty acids, dietary sterols, fat-soluble vitamins, and dietary sphingolipids in the diet may be a promising nutritional strategy to reduce the risk of T2DM through the mechanisms specified. However, it can be said that there are still few studies on the relationship between lipidomics and the gut-brain axis in T2DM because lipidomics are relatively new technology products. It is yet early to decide on the uses of lipidomics in diet plans in T2DMs. So, future investigations should be addressed to clarify the relationship between diet, gut microbiota and lipidomics in T2DM. Examining the these dietary components in future studies in different model morganisms and clinical trials at different doses and times will help to reveal possible relationships.

## Data Availability

Not applicable.

## Abbreviations

1,25(OH)_2_D: 1,25-Dihydroxyvitamin D
24,25(OH)_2_D: 24,25-Dihydroxyvitamin D
5-HT: Serotonin
AA: Arachidonic acid
ABCA1: ATP-binding cassette transporter A1
ACC1: Acetyl-CoA carboxylases 1
ACLY: Activating ATP-citrate lyase
ALA: Alpha-linoleic acid
AMPK: AMP Kinase
Apo: Apolipoprotein
CD14: Cluster-of-differentiation 14
CETP: Cholesteryl ester transfer protein
ChREBP: Carbohydrate response element-binding protein
CNS: Central nervous system
COX: Cyclooxygenases
DHA: Docosahexaenoic acid
DM: Diabetes
DNL: De novo lipogenesis
EEC: Enteroendocrine cells
ENS: Enteric nervous system
EPA: Eicosapentaenoic acid
ER: Endoplasmic reticulum
FAs: Fatty acids
FADS1: Fatty acid desaturases 1
FADS2: Fatty acid desaturases 2
FAK: Focal adhesion kinase
FAO: Food and Agriculture Organization
FASN: Fatty acid synthase
FFA: Free fatty acids
FXR: Farnesoid X receptor
GF: Germ-free
GLP-1: Glucagon-like peptide-1
GPR40: G proteincoupled receptor 40
HDL: High dense lipoprotein cholesterol
HSPGs: Heparan sulfate proteoglycans
ICAM-1: Intercellular adhesion molecule-1
IL: Interleukin
INFγ: Interferon-γ
iNOS: Inducible nitric oxide synthase
IR: Insulin resistance
IRAK4: IL-1R-associated kinase 4
IRS-1: Insulin receptor substrate-1
LA: Linoleic acid
LBP: Lipopolysaccharide-binding proteins
LCAT: Lecithin cholesterol acyl transferase
LDL: Low density lipoprotein cholesterol
LOX: Lipoxygenases
LPL: Lipoprotein lipase
LPS: Lipopolysaccharides
LRP: LDL receptor-associated protein
MAPK: Mitogen-activated protein kinase
METSIM: Metabolic Syndrome in Men
MLX: Insulin activates Max-like protein X
MM-LDL: Minimally oxidized LDL
mRNA: Messenger RNA
MTP: Microsomal triglyceride transfer protein
MUFA: Monounsaturated fatty acids
NCDs: Noncommunicable diseases
NEFA: Non-esterified fatty acids
NPC1L1: Niemann-Pick C1-like 1 protein
Ox-LDL: Fully oxidized LDL
PGC1α: Peroxisome proliferator-activated receptor co-activator-1α
PIP2: Phosphatidylinositol 4,5-biphosphate
PIP3: Phosphatidylinositol 3,4,5-triphosphate
PKA: Protein kinase A
PKB: Protein kinase B
PPAR: Peroxisome proliferator-activated receptor
PUFA: Polyunsaturated fatty acids
ROS: Reactive oxygen species
SCD1: Stearoyl-CoA desaturase-1
SCFA: Short-chain fatty acids
SFA: Saturated fatty acids
SIRT1: Sirtuin1
SPMs: Specialized pro-resolving mediators
SR-B1: Scavenger receptor B1
T2DM: Type 2 diabetes mellitus
TC: Total cholesterol
TCA: Tricarboxylic acid cycle
TFA: Trans fatty acids
TG: Triglyceride
TGR5: Takeda-G-protein-receptor-5
TLR-4: Toll-Like Receptor 4
TNFR1: TNF-receptor 1
TNFR2: TNF-receptor 2
TNF-α: Tumor necrosis factor alpha
TRLs: Triglyceride-rich lipoproteins
VLDL: Very low-density lipoprotein
WAT: White adipose tissue
δTE: δ-Tocotrienol

## References

[1] Roden M, Shulman GI (2019). The integrative biology of type 2 diabetes. Nature.

[2] Chatterjee S, Khunti K, Davies MJ (2017). Type 2 diabetes. The Lancet.

[3] Paulson KR, Kamath AM, Alam T, Bienhoff K, Abady GG, Abbas J (2021). Global, regional, and national progress towards Sustainable Development Goal 3.2 for neonatal and child health: all-cause and cause-specific mortality findings from the Global Burden of Disease Study 2019. Lancet.

[4] American Diabetes Association Professional Practice Committee (2022). 2. Classification and diagnosis of diabetes: Standards of medical care in diabetes—2022. Diabetes Care..

[5] International Diabetes Federation. IDF Diabetes Atlas 2021. https://diabetesatlas.org/atlas/tenth-edition/ Accessed 11 November 2022

[6] Galicia-Garcia U, Benito-Vicente A, Jebari S, Larrea-Sebal A, Siddiqi H, Uribe KB (2020). Pathophysiology of type 2 diabetes mellitus. Int J Mol Sci.

[7] Schellenberg ES, Dryden DM, Vandermeer B, Ha C, Korownyk C (2013). Lifestyle interventions for patients with and at risk for type 2 diabetes: a systematic review and meta-analysis. Ann Intern Med.

[8] Chan JC, Lim L-L, Wareham NJ, Shaw JE, Orchard TJ, Zhang P (2020). The Lancet Commission on diabetes: using data to transform diabetes care and patient lives. Lancet.

[9] Mahajan A, Taliun D, Thurner M, Robertson NR, Torres JM, Rayner NW (2018). Fine-mapping type 2 diabetes loci to single-variant resolution using high-density imputation and islet-specific epigenome maps. Nat Genet.

[10] Srinivasan S, Chen L, Todd J, Divers J, Gidding S, Chernausek S, ProDiGY Consortium (2021). The First Genome-Wide Association Study for Type 2 Diabetes in Youth The Progress in Diabetes Genetics in Youth (ProDiGY) Consortium. Diabetes..

[11] Athyros VG, Doumas M, Imprialos KP, Stavropoulos K, Georgianou E, Katsimardou A (2018). Diabetes and lipid metabolism. Hormones.

[12] Ljubkovic M, Gressette M, Bulat C, Cavar M, Bakovic D, Fabijanic D (2019). Disturbed fatty acid oxidation, endoplasmic reticulum stress, and apoptosis in left ventricle of patients with type 2 diabetes. Diabetes.

[13] Eid S, Sas KM, Abcouwer SF, Feldman EL, Gardner TW, Pennathur S (2019). New insights into the mechanisms of diabetic complications: role of lipids and lipid metabolism. Diabetologia.

[14] Huynh K, Martins RN, Meikle PJ (2017). Lipidomic profiles in diabetes and dementia. J Alzheimers Dis.

[15] Wishart DS (2019). Metabolomics for investigating physiological and pathophysiological processes. Physiol Rev.

[16] Postle AD (2012). Lipidomics. Curr Opin Clin Nutr Metab Care.

[17] Gonzalez-Covarrubias V (2013). Lipidomics in longevity and healthy aging. Biogerontology.

[18] Li J, Xie H, Li A, Cheng J, Yang K, Wang J (2017). Distinct plasma lipids profiles of recurrent ovarian cancer by liquid chromatography-mass spectrometry. Oncotarget.

[19] Varma VR, Oommen AM, Varma S, Casanova R, An Y, Andrews RM (2018). Brain and blood metabolite signatures of pathology and progression in Alzheimer disease: a targeted metabolomics study. PLoS Med.

[20] Vvedenskaya O, Rose TD, Knittelfelder O, Palladini A, Wodke JAH, Schuhmann K (2021). Nonalcoholic fatty liver disease stratification by liver lipidomics. J Lipid Res.

[21] Markgraf DF, Al-Hasani H, Lehr S (2016). Lipidomics—Reshaping the analysis and perception of type 2 diabetes. Int J Mol Sci.

[22] Alshehry ZH, Mundra PA, Barlow CK, Mellett NA, Wong G, McConville MJ (2016). Plasma lipidomic profiles improve on traditional risk factors for the prediction of cardiovascular events in type 2 diabetes mellitus. Circulation.

[23] Yun H, Sun L, Wu Q, Zong G, Qi Q, Li H (2020). Associations among circulating sphingolipids, β-cell function, and risk of developing type 2 diabetes: a population-based cohort study in China. PLoS Med.

[24] Lu J, Lam SM, Wan Q, Shi L, Huo Y, Chen L (2019). High-coverage targeted lipidomics reveals novel serum lipid predictors and lipid pathway dysregulation antecedent to type 2 diabetes onset in normoglycemic Chinese adults. Diabetes Care.

[25] Rhee EP, Cheng S, Larson MG, Walford GA, Lewis GD, McCabe E (2011). Lipid profiling identifies a triacylglycerol signature of insulin resistance and improves diabetes prediction in humans. J Clin Investig.

[26] Haus JM, Kashyap SR, Kasumov T, Zhang R, Kelly KR, DeFronzo RA (2009). Plasma ceramides are elevated in obese subjects with type 2 diabetes and correlate with the severity of insulin resistance. Diabetes.

[27] Wang-Sattler R, Yu Z, Herder C, Messias AC, Floegel A, He Y (2012). Novel biomarkers for pre-diabetes identified by metabolomics. Mol Syst Biol.

[28] Boon J, Hoy AJ, Stark R, Brown RD, Meex RC, Henstridge DC (2013). Ceramides contained in LDL are elevated in type 2 diabetes and promote inflammation and skeletal muscle insulin resistance. Diabetes.

[29] Bartelt A, Koehne T, Tödter K, Reimer R, Müller B, Behler-Janbeck F (2017). Quantification of bone fatty acid metabolism and its regulation by adipocyte lipoprotein lipase. Int J Mol Sci.

[30] Food and Agriculture Organization of the United Nations (2010). Fats and fatty acids in human nutrition: report of an expert consultation. FAO Food Nutr Pap.

[31] Perona JS (2017). Membrane lipid alterations in the metabolic syndrome and the role of dietary oils. Biochim Biophys Acta Biomembr..

[32] Risérus U (2008). Fatty acids and insulin sensitivity. Curr Opin Clin Nutr Metab Care.

[33] Small L, Brandon AE, Turner N, Cooney GJ (2018). Modeling insulin resistance in rodents by alterations in diet: what have high-fat and high-calorie diets revealed?. Am J Physiol Endocrinol Metab.

[34] Sampath H, Ntambi JM (2005). Polyunsaturated fatty acid regulation of genes of lipid metabolism. Annu Rev Nutr.

[35] Bays HE, Tighe AP, Sadovsky R, Davidson MH (2008). Prescription omega-3 fatty acids and their lipid effects: physiologic mechanisms of action and clinical implications. Expert Rev Cardiovasc Ther.

[36] Vannice G, Rasmussen H (2014). Position of the academy of nutrition and dietetics: dietary fatty acids for healthy adults. J Acad Nutr Diet.

[37] Clarke SD, Jump DΒ (2018). Regulation of hepatic gene expression by dietary fats: a unique role for polyunsaturated fatty acids.

[38] Food and Agriculture Organization of the United Nations. Fats and fatty acids in human nutrition: report of an expert consultation. FAO Food and Nutrition Paper. 2008. 21812367

[39] Mancini A, Imperlini E, Nigro E, Montagnese C, Daniele A, Orrù S (2015). Biological and nutritional properties of palm oil and palmitic acid: effects on health. Molecules.

[40] Risérus U, Willett WC, Hu FB (2009). Dietary fats and prevention of type 2 diabetes. Prog Lipid Res.

[41] Siemelink M, Verhoef A, Dormans J, Span P, Piersma A (2002). Dietary fatty acid composition during pregnancy and lactation in the rat programs growth and glucose metabolism in the offspring. Diabetologia.

[42] Golson M, Misfeldt AA, Kopsombut U, Petersen C, Gannon M (2010). High fat diet regulation of β-cell proliferation and β-cell mass. Open Endocrinol J.

[43] Meli R, Mattace Raso G, Irace C, Simeoli R, Di Pascale A, Paciello O (2013). High fat diet induces liver steatosis and early dysregulation of iron metabolism in rats. PLoS ONE.

[44] Muoio DM, Newgard CB (2008). Fatty acid oxidation and insulin action: when less is more. Diabetes.

[45] Prasad M, Rajagopal P, Devarajan N, Veeraraghavan VP, Palanisamy CP, Cui B (2022). A comprehensive review on high fat diet-induced diabetes mellitus: an epigenetic view. J Nutr Biochem.

[46] Micha R, Mozaffarian D (2010). Saturated fat and cardiometabolic risk factors, coronary heart disease, stroke, and diabetes: a fresh look at the evidence. Lipids.

[47] Julibert A, Bibiloni MdM, Bouzas C, Martínez-González MÁ, Salas-Salvadó J, Corella D (2019). Total and subtypes of dietary fat intake and its association with components of the metabolic syndrome in a mediterranean population at high cardiovascular risk. Nutrients..

[48] Abshirini M, Mahaki B, Bagheri F, Siassi F, Koohdani F, Qorbani M (2020). Dietary fat quality and pre-diabetes: a case-control study. Int J Prev Med.

[49] Yubero-Serrano EM, Delgado-Lista J, Tierney AC, Perez-Martinez P, Garcia-Rios A, Alcala-Diaz JF (2015). Insulin resistance determines a differential response to changes in dietary fat modification on metabolic syndrome risk factors: the LIPGENE study. Am J Clin Nutr.

[50] Imamura F, Micha R, Wu JH, de Oliveira Otto MC, Otite FO, Abioye AI (2016). Effects of saturated fat, polyunsaturated fat, monounsaturated fat, and carbohydrate on glucose-insulin homeostasis: a systematic review and meta-analysis of randomised controlled feeding trials. PLoS Med.

[51] Gaeini Z, Bahadoran Z, Mirmiran P (2022). Saturated fatty acid intake and risk of type 2 diabetes: an updated systematic review and dose-response meta-analysis of cohort studies. Adv Nutr.

[52] Liu S, van der Schouw YT, Soedamah-Muthu SS, Spijkerman AM, Sluijs I (2019). Intake of dietary saturated fatty acids and risk of type 2 diabetes in the European Prospective Investigation into Cancer and Nutrition-Netherlands cohort: associations by types, sources of fatty acids and substitution by macronutrients. Eur J Nutr.

[53] Morio B, Fardet A, Legrand P, Lecerf J-M (2016). Involvement of dietary saturated fats, from all sources or of dairy origin only, in insulin resistance and type 2 diabetes. Nutr Rev.

[54] EFSA Panel on Dietetic Products N, Allergies (2010). Scientific opinion on dietary reference values for fats, including saturated fatty acids, polyunsaturated fatty acids, monounsaturated fatty acids, trans fatty acids, and cholesterol. EFSA J.

[55] Islam MA, Amin MN, Siddiqui SA, Hossain MP, Sultana F, Kabir MR (2019). Trans fatty acids and lipid profile: a serious risk factor to cardiovascular disease, cancer and diabetes. Diabetes Metab Syndr.

[56] Pipoyan D, Stepanyan S, Stepanyan S, Beglaryan M, Costantini L, Molinari R (2021). The effect of trans fatty acids on human health: regulation and consumption patterns. Foods.

[57] Dorfman SE, Laurent D, Gounarides JS, Li X, Mullarkey TL, Rocheford EC (2009). Metabolic implications of dietary trans-fatty acids. Obesity.

[58] Menaa F, Menaa A, Menaa B, Tréton J (2013). Trans-fatty acids, dangerous bonds for health? A background review paper of their use, consumption, health implications and regulation in France. Eur J Nutr.

[59] Lopez S, Bermudez B, Ortega A, Varela LM, Pacheco YM, Villar J (2011). Effects of meals rich in either monounsaturated or saturated fat on lipid concentrations and on insulin secretion and action in subjects with high fasting triglyceride concentrations. Am J Clin Nutr.

[60] Bhaswant M, Poudyal H, Brown L (2015). Mechanisms of enhanced insulin secretion and sensitivity with n-3 unsaturated fatty acids. J Nutr Biochem.

[61] Tierney AC, Roche HM (2007). The potential role of olive oil-derived MUFA in insulin sensitivity. Mol Nutr Food Res.

[62] Bulotta S, Celano M, Lepore SM, Montalcini T, Pujia A, Russo D (2014). Beneficial effects of the olive oil phenolic components oleuropein and hydroxytyrosol: focus on protection against cardiovascular and metabolic diseases. J Transl Med.

[63] Dreher ML, Davenport AJ (2013). Hass avocado composition and potential health effects. Crit Rev Food Sci Nutr.

[64] Andersson-Hall U, Carlsson N-G, Sandberg A-S, Holmäng A (2018). Circulating linoleic acid is associated with improved glucose tolerance in women after gestational diabetes. Nutrients.

[65] Di Pasquale MG (2009). The essentials of essential fatty acids. J Diet Suppl.

[66] Shetty SS, Kumari NS, Varadarajan R (2023). The ratio of omega-6/omega-3 fatty acid: implications and application as a marker to diabetes. Biomarkers Diabetes..

[67] Brown TJ, Brainard J, Song F, Wang X, Abdelhamid A, Hooper L (2019). Omega-3, omega-6, and total dietary polyunsaturated fat for prevention and treatment of type 2 diabetes mellitus: systematic review and meta-analysis of randomised controlled trials. BMJ.

[68] Simopoulos AP (2014). The impact of the Bellagio report on healthy agriculture, healthy nutrition, healthy people: scientific and policy aspects and the international network of centers for genetics, nutrition and fitness for health. Lifestyle Genomics.

[69] Simopoulos AP (2009). Omega-6/omega-3 essential fatty acids: biological effects. World Rev Nutr Diet.

[70] Shetty SS, Shetty PK (2020). ω-6/ω-3 fatty acid ratio as an essential predictive biomarker in the management of type 2 diabetes mellitus. Nutrition.

[71] Wu Y, Ding Y, Tanaka Y, Zhang W (2014). Risk factors contributing to type 2 diabetes and recent advances in the treatment and prevention. Int J Med Sci.

[72] Day EA, Ford RJ, Steinberg GR (2017). AMPK as a therapeutic target for treating metabolic diseases. Trends Endocrinol Metab.

[73] Pérez-Matute P, Pérez-Echarri N, Martínez JA, Marti A, Moreno-Aliaga MJ (2007). Eicosapentaenoic acid actions on adiposity and insulin resistance in control and high-fat-fed rats: role of apoptosis, adiponectin and tumour necrosis factor-α. Br J Nutr.

[74] Vessby B, Ahrén B, Warensjö E, Lindgärde F (2012). Plasma lipid fatty acid composition, desaturase activities and insulin sensitivity in Amerindian women. Nutr Metab Cardiovasc Dis.

[75] Talukdar S, Bae EJ, Imamura T, Morinaga H, Fan W, Li P (2010). GPR120 is an omega-3 fatty acid receptor mediating potent anti-inflammatory and insulin-sensitizing effects. Cell.

[76] Sarbolouki S, Javanbakht MH, Derakhshanian H, Hosseinzadeh P, Zareei M, Hashemi SB (2013). Eicosapentaenoic acid improves insulin sensitivity and blood sugar in overweight type 2 diabetes mellitus patients: a double-blind randomised clinical trial. Singapore Med J.

[77] Zheng JS, Lin M, Fang L, Yu Y, Yuan L, Jin Y (2016). Effects of n-3 fatty acid supplements on glycemic traits in Chinese type 2 diabetic patients: a double-blind randomized controlled trial. Mol Nutr Food Res.

[78] Pooya S, Jalali MD, Jazayery AD, Saedisomeolia A, Eshraghian MR, Toorang F (2010). The efficacy of omega-3 fatty acid supplementation on plasma homocysteine and malondialdehyde levels of type 2 diabetic patients. Nutr Metab Cardiovasc Dis.

[79] Rylander C, Sandanger TM, Engeset D, Lund E (2014). Consumption of lean fish reduces the risk of type 2 diabetes mellitus: a prospective population based cohort study of Norwegian women. PLoS ONE.

[80] Tørris C, Molin M, Småstuen MC (2017). Lean fish consumption is associated with beneficial changes in the metabolic syndrome components: a 13-year follow-up study from the Norwegian Tromsø study. Nutrients.

[81] Øyen J, Madsen L, Brantsæter AL, Skurtveit SO, Egeland GM (2019). Lean fish intake decreases the risk of type 2 diabetes mellitus in Norwegian Women (P18–036–19). Curr Dev Nutr.

[82] Liaset B, Øyen J, Jacques H, Kristiansen K, Madsen L (2019). Seafood intake and the development of obesity, insulin resistance and type 2 diabetes. Nutr Res Rev.

[83] Yary T, Voutilainen S, Tuomainen T-P, Ruusunen A, Nurmi T, Virtanen JK (2016). Serum n–6 polyunsaturated fatty acids, Δ 5-and Δ 6-desaturase activities, and risk of incident type 2 diabetes in men: the Kuopio Ischaemic Heart Disease Risk Factor Study. Am J Clin Nutr.

[84] Mansouri V, Javanmard SH, Mahdavi M, Tajedini MH (2018). Association of polymorphism in fatty acid desaturase gene with the risk of Type 2 diabetes in iranian population. Adv Biomed Res.

[85] Wang X, Chan CB (2015). n-3 polyunsaturated fatty acids and insulin secretion. J Endocrinol.

[86] Wang F, Wang Y, Zhu Y, Liu X, Xia H, Yang X (2017). Treatment for 6 months with fish oil-derived n-3 polyunsaturated fatty acids has neutral effects on glycemic control but improves dyslipidemia in type 2 diabetic patients with abdominal obesity: a randomized, double-blind, placebo-controlled trial. Eur J Nutr.

[87] Balfegó M, Canivell S, Hanzu FA, Sala-Vila A, Martínez-Medina M, Murillo S (2016). Effects of sardine-enriched diet on metabolic control, inflammation and gut microbiota in drug-naïve patients with type 2 diabetes: a pilot randomized trial. Lipids Health Dis.

[88] Crochemore ICC, Souza AF, de Souza AC, Rosado EL (2012). ω-3 polyunsaturated fatty acid supplementation does not influence body composition, insulin resistance, and lipemia in women with type 2 diabetes and obesity. Nutr Clin Pract.

[89] Wu JH, Marklund M, Imamura F, Tintle N, Korat AVA, De Goede J (2017). Omega-6 fatty acid biomarkers and incident type 2 diabetes: pooled analysis of individual-level data for 39 740 adults from 20 prospective cohort studies. Lancet Diabetes Endocrinol.

[90] Forouhi NG, Imamura F, Sharp SJ, Koulman A, Schulze MB, Zheng J (2016). Association of plasma phospholipid n-3 and n-6 polyunsaturated fatty acids with type 2 diabetes: the EPIC-InterAct case-cohort study. PLoS Med.

[91] Weir NL, Nomura SO, Steffen BT, Guan W, Karger AB, Klein R (2020). Associations between omega-6 polyunsaturated fatty acids, hyperinsulinemia and incident diabetes by race/ethnicity: the multi-ethnic study of atherosclerosis. Clin Nutr.

[92] Vessby B, Uusitupa M, Hermansen K, Riccardi G, Rivellese AA, Tapsell LC (2001). Substituting dietary saturated for monounsaturated fat impairs insulin sensitivity in healthy men and women: the KANWU study. Diabetologia.

[93] Jebb SA, Lovegrove JA, Griffin BA, Frost GS, Moore CS, Chatfield MD, Bluck LJ, Williams CM, Sanders TA, RISCK Study Group (2010). Effect of changing the amount and type of fat and carbohydrate on insulin sensitivity and cardiovascular risk: the RISCK (Reading, Imperial, Surrey, Cambridge, and Kings) trial. Am J Clin Nutr.

[94] Tierney AC, McMonagle J, Shaw D, Gulseth H, Helal O, Saris W (2011). Effects of dietary fat modification on insulin sensitivity and on other risk factors of the metabolic syndrome—LIPGENE: a European randomized dietary intervention study. Int J Obes.

[95] Bjermo H, Iggman D, Kullberg J, Dahlman I, Johansson L, Persson L (2012). Effects of n-6 PUFAs compared with SFAs on liver fat, lipoproteins, and inflammation in abdominal obesity: a randomized controlled trial. Am J Clin Nutr.

[96] Rosqvist F, Iggman D, Kullberg J, Cedernaes J, Johansson H-E, Larsson A (2014). Overfeeding polyunsaturated and saturated fat causes distinct effects on liver and visceral fat accumulation in humans. Diabetes.

[97] Papatheodorou K, Papanas N, Banach M, Papazoglou D, Edmonds M (2016). Complications of Diabetes 2016. J Diabetes Res.

[98] Dalfrà MG, Burlina S, Del Vescovo GG, Lapolla A (2020). Genetics and epigenetics: new insight on gestational diabetes mellitus. Front Endocrinol.

[99] Magkos F, Wang X, Mittendorfer B (2010). Metabolic actions of insulin in men and women. Nutrition.

[100] Burgos-Morón E, Abad-Jiménez Z, Martínez de Marañón A, Iannantuoni F, Escribano-López I, López-Domènech S (2019). Relationship between oxidative stress, ER stress, and inflammation in type 2 diabetes: the battle continues. J Clin Med.

[101] Alves-Bezerra M, Cohen DE (2017). Triglyceride Metabolism in the Liver. Compr Physiol.

[102] Birkenfeld AL, Shulman GI (2014). Nonalcoholic fatty liver disease, hepatic insulin resistance, and type 2 diabetes. Hepatology.

[103] Schoeler M, Caesar R (2019). Dietary lipids, gut microbiota and lipid metabolism. Rev Endocr Metab Disord.

[104] Jovandaric MZ, Milenkovic SJ. 2020. Significance of lipid and lipoprotein in organism. Apolipoproteins, Triglycerides and Cholesterol: IntechOpen.

[105] Lairon D (2009). Digestion and absorption of lipids.

[106] Amara S, Bourlieu C, Humbert L, Rainteau D, Carrière F (2019). Variations in gastrointestinal lipases, pH and bile acid levels with food intake, age and diseases: Possible impact on oral lipid-based drug delivery systems. Adv Drug Deliv Rev.

[107] Hornbuckle WE, Tennant BC (1997). Gastrointestinal function.

[108] Hussain MM (2014). Intestinal lipid absorption and lipoprotein formation. Curr Opin Lipidol.

[109] Kindel T, Lee DM, Tso P (2010). The mechanism of the formation and secretion of chylomicrons. Atheroscler Suppl.

[110] Kumari A, Kristensen KK, Ploug M, Winther A-ML (2021). The importance of lipoprotein lipase regulation in atherosclerosis. Biomedicines..

[111] Goldberg IJ, Eckel RH, Abumrad NA (2009). Regulation of fatty acid uptake into tissues: lipoprotein lipase-and CD36-mediated pathways. J Lipid Res.

[112] Morita S-y (2016). Metabolism and modification of apolipoprotein B-containing lipoproteins involved in dyslipidemia and atherosclerosis. Biol Pharm Bull.

[113] Roslan Z, Muhamad M, Selvaratnam L, Ab-Rahim S (2019). The roles of low-density lipoprotein receptor-related proteins 5, 6, and 8 in cancer: a review. J Oncol.

[114] Haas ME, Attie AD, Biddinger SB (2013). The regulation of ApoB metabolism by insulin. Trends Endocrinol Metab.

[115] Au DT, Strickland DK, Muratoglu SC (2017). The LDL receptor-related protein 1: at the crossroads of lipoprotein metabolism and insulin signaling. J Diabetes Res.

[116] Kamagate A, Dong HH (2008). FoxO1 integrates insulin signaling to VLDL production. Cell Cycle.

[117] Allister EM, Borradaile NM, Edwards JY, Huff MW (2005). Inhibition of microsomal triglyceride transfer protein expression and apolipoprotein B100 secretion by the citrus flavonoid naringenin and by insulin involves activation of the mitogen-activated protein kinase pathway in hepatocytes. Diabetes.

[118] Feingold KR. Introduction to lipids and lipoproteins. In: Feingold KR, Anawalt B, Blackman MR, et al., editors. Endotext. South Dartmouth (MA):MDText.com, Inc., 2000-. https://www.ncbi.nlm.nih.gov/books/NBK305896/. Accessed 19 Jan 2021

[119] Chan DC, Watts GF, Nguyen MN, Barrett PHR (2006). Factorial study of the effect of n–3 fatty acid supplementation and atorvastatin on the kinetics of HDL apolipoproteins AI and A-II in men with abdominal obesity. Am J Clin Nutr.

[120] Bonizzi A, Piuri G, Corsi F, Cazzola R, Mazzucchelli S (2021). HDL dysfunctionality: clinical relevance of quality rather than quantity. Biomedicines.

[121] Chapman MJ, Le Goff W, Guerin M, Kontush A (2010). Cholesteryl ester transfer protein: at the heart of the action of lipid-modulating therapy with statins, fibrates, niacin, and cholesteryl ester transfer protein inhibitors. Eur Heart J.

[122] Fossati P, Romon-Rousseaux M (1987). Insulin and HDL-cholesterol metabolism. Diabete Metabolisme.

[123] Song Z, Xiaoli AM, Yang F (2018). Regulation and metabolic significance of de novo lipogenesis in adipose tissues. Nutrients.

[124] Samuel VT, Shulman GI (2016). The pathogenesis of insulin resistance: integrating signaling pathways and substrate flux. J Clin Investig.

[125] Parhofer KG (2015). Interaction between glucose and lipid metabolism: more than diabetic dyslipidemia. Diabetes Metab J.

[126] Visser J, van Zwol W, Kuivenhoven JA (2022). Managing of dyslipidaemia characterized by accumulation of triglyceride-rich lipoproteins. Current Atheroscler Rep.

[127] Nogueira J-P, Maraninchi M, Béliard S, Padilla N, Duvillard L, Mancini J (2012). Absence of acute inhibitory effect of insulin on chylomicron production in type 2 diabetes. Arterioscler Thromb Vasc Biol.

[128] Hong D-Y, Lee D-H, Lee J-Y, Lee E-C, Park S-W, Lee M-R (2022). Relationship between brain metabolic disorders and cognitive impairment: LDL receptor defect. Int J Mol Sci.

[129] Hsieh J, Hayashi AA, Webb J, Adeli K (2008). Postprandial dyslipidemia in insulin resistance: mechanisms and role of intestinal insulin sensitivity. Atheroscler Suppl.

[130] Xiao C, Lewis GF (2012). Regulation of chylomicron production in humans. Biochim Biophys Acta Mol Cell Biol Lipids..

[131] Hirano T (2018). Pathophysiology of diabetic dyslipidemia. J Atheroscler Thromb.

[132] Verges B (2009). Lipid modification in type 2 diabetes: the role of LDL and HDL. Fundam Clin Pharmacol.

[133] Duvillard L, Florentin E, Lizard G, Petit J-M, Galland F, Monier S (2003). Cell surface expression of LDL receptor is decreased in type 2 diabetic patients and is normalized by insulin therapy. Diabetes Care.

[134] Grosjean A, Venteclef N, Dalmas E (2021). Understanding the heterogeneity and functions of metabolic tissue macrophages. Semin Cell Dev Biol.

[135] Kolliniati O, Ieronymaki E, Vergadi E, Tsatsanis C (2022). Metabolic regulation of macrophage activation. J Innate Immun.

[136] Püschel GP, Klauder J, Henkel J (2022). Macrophages, low-grade inflammation, insulin resistance and hyperinsulinemia: a mutual ambiguous relationship in the development of metabolic diseases. J Clin Med.

[137] Su D, Coudriet GM, Hyun Kim D, Lu Y, Perdomo G, Qu S (2009). FoxO1 links insulin resistance to proinflammatory cytokine IL-1β production in macrophages. Diabetes.

[138] Bonilha I, Hajduch E, Luchiari B, Nadruz W, Le Goff W, Sposito AC (2021). The reciprocal relationship between LDL metabolism and type 2 diabetes mellitus. Metabolites.

[139] Zingg J-M, Vlad A, Ricciarelli R (2021). Oxidized ldls as signaling molecules. Antioxidants.

[140] Prieur X, Rőszer T, Ricote M (2010). Lipotoxicity in macrophages: evidence from diseases associated with the metabolic syndrome. Biochim Biophys Acta Mol Cell Biol Lipids..

[141] Lombardo YB, Chicco AG (2006). Effects of dietary polyunsaturated n-3 fatty acids on dyslipidemia and insulin resistance in rodents and humans. A review. J Nutr Biochem.

[142] Ooi EM, Watts GF, Ng TW, Barrett PHR (2015). Effect of dietary fatty acids on human lipoprotein metabolism: a comprehensive update. Nutrients.

[143] Wong AT, Chan DC, Barrett PHR, Adams LA, Watts GF (2014). Effect of ω-3 fatty acid ethyl esters on apolipoprotein B-48 kinetics in obese subjects on a weight-loss diet: a new tracer kinetic study in the postprandial state. J Clin Endocrinol Metab.

[144] Wang J-f, Zhang H-m, Li Y-y, Xia S, Wei Y, Yang L (2019). A combination of omega-3 and plant sterols regulate glucose and lipid metabolism in individuals with impaired glucose regulation: a randomized and controlled clinical trial. Lipids Health Dis.

[145] Jump DB (2011). Fatty acid regulation of hepatic lipid metabolism. Curr Opin Clin Nutr Metab Care.

[146] Stupin M, Kibel A, Stupin A, Selthofer-Relatić K, Matić A, Mihalj M (2019). The physiological effect of n-3 polyunsaturated fatty acids (n-3 PUFAs) intake and exercise on hemorheology, microvascular function, and physical performance in health and cardiovascular diseases; Is there an interaction of exercise and dietary n-3 PUFA intake?. Front Physiol.

[147] Cisa-Wieczorek S, Hernández-Alvarez MI (2020). Deregulation of lipid homeostasis: a Fa (c) t in the development of metabolic diseases. Cells.

[148] Devkota S, Wang Y, Musch MW, Leone V, Fehlner-Peach H, Nadimpalli A (2012). Dietary-fat-induced taurocholic acid promotes pathobiont expansion and colitis in Il10−/− mice. Nature.

[149] Fritzen AM, Lundsgaard A-M, Kiens B (2020). Tuning fatty acid oxidation in skeletal muscle with dietary fat and exercise. Nat Rev Endocrinol.

[150] Briaud I, Kelpe CL, Johnson LM, Tran POT, Poitout V (2002). Differential effects of hyperlipidemia on insulin secretion in islets of langerhans from hyperglycemic versus normoglycemic rats. Diabetes.

[151] Ahima RS, Lazar MA (2008). Adipokines and the peripheral and neural control of energy balance. Mol Endocrinol.

[152] Li M, Chi X, Wang Y, Setrerrahmane S, Xie W, Xu H (2022). Trends in insulin resistance: insights into mechanisms and therapeutic strategy. Signal Transduct Target Ther.

[153] Lee C-Y, Lee C-H, Tsai S, Huang C-T, Wu M-T, Tai S-Y (2009). Association between serum leptin and adiponectin levels with risk of insulin resistance and impaired glucose tolerance in non-diabetic women. Kaohsiung J Med Sci.

[154] Abdel-Moneim A, Abd El-Twab SM, Yousef AI, Reheim ESA, Ashour MB (2018). Modulation of hyperglycemia and dyslipidemia in experimental type 2 diabetes by gallic acid and p-coumaric acid: The role of adipocytokines and PPARγ. Biomed Pharmacother.

[155] Dilworth L, Facey A, Omoruyi F (2021). Diabetes mellitus and its metabolic complications: the role of adipose tissues. Int J Mol Sci.

[156] Lago F, Gómez R, Gómez-Reino JJ, Dieguez C, Gualillo O (2009). Adipokines as novel modulators of lipid metabolism. Trends Biochem Sci.

[157] Christou G, Kiortsis D (2013). Adiponectin and lipoprotein metabolism. Obes Rev.

[158] Bjornstad P, Eckel RH (2018). Pathogenesis of lipid disorders in insulin resistance: a brief review. Curr DiabRep.

[159] Blüher M (2013). Importance of adipokines in glucose homeostasis. Diabetes Management.

[160] Jiang S, Young JL, Wang K, Qian Y, Cai L (2020). Diabetic-induced alterations in hepatic glucose and lipid metabolism: the role of type 1 and type 2 diabetes mellitus. Mol Med Rep.

[161] Hyötyläinen T, Bondia-Pons I, Orešič M (2013). Lipidomics in nutrition and food research. Mol Nutr Food Res.

[162] Moffa S, Mezza T, Cefalo C, Cinti F, Impronta F, Sorice GP (2019). The interplay between immune system and microbiota in diabetes. Mediators Inflamm.

[163] Bocanegra A, Macho-González A, Garcimartín A, Benedí J, Sánchez-Muniz FJ (2021). Whole alga, algal extracts, and compounds as ingredients of functional foods: composition and action mechanism relationships in the prevention and treatment of type-2 diabetes mellitus. Int J Mol Sci.

[164] Chung H-J, Sim J-H, Min T-S, Choi H-K (2018). Metabolomics and lipidomics approaches in the science of probiotics: a review. J Med Food.

[165] Shetty SS, Kumari S (2021). Fatty acids and their role in type-2 diabetes. Exp Ther Med.

[166] Wang S, Yong H, He X-D (2021). Multi-omics: Opportunities for research on mechanism of type 2 diabetes mellitus. World J Diabetes.

[167] Bessac A, Cani PD, Meunier E, Dietrich G, Knauf C (2018). Inflammation and gut-brain axis during type 2 diabetes: focus on the crosstalk between intestinal immune cells and enteric nervous system. Front Neurosci.

[168] Wachsmuth HR, Weninger SN, Duca FA (2022). Role of the gut–brain axis in energy and glucose metabolism. Exp Mol Med.

[169] Richards P, Thornberry NA, Pinto S (2021). The gut–brain axis: Identifying new therapeutic approaches for type 2 diabetes, obesity, and related disorders. Mol Metab.

[170] Rastelli M, Knauf C, Cani PD (2018). Gut microbes and health: a focus on the mechanisms linking microbes, obesity, and related disorders. Obesity.

[171] Migrenne S, Marsollier N, Cruciani-Guglielmacci C, Magnan C (2006). Importance of the gut–brain axis in the control of glucose homeostasis. Curr Opin Pharmacol.

[172] Lew KN, Starkweather A, Cong X, Judge M (2019). A Mechanistic model of gut-brain axis perturbation and high-fat diet pathways to gut microbiome homeostatic disruption, systemic inflammation, and type 2 diabetes. Biol Res Nurs.

[173] Ussar S, Fujisaka S, Kahn CR (2016). Interactions between host genetics and gut microbiome in diabetes and metabolic syndrome. Mol Metab.

[174] Bäckhed F, Ding H, Wang T, Hooper LV, Koh GY, Nagy A (2004). The gut microbiota as an environmental factor that regulates fat storage. Proc Natl Acad Sci.

[175] Cani PD, Knauf C, Iglesias MA, Drucker DJ, Delzenne NM, Burcelin R (2006). Improvement of glucose tolerance and hepatic insulin sensitivity by oligofructose requires a functional glucagon-like peptide 1 receptor. Diabetes.

[176] Utzschneider KM, Kratz M, Damman CJ, Hullarg M (2016). Mechanisms linking the gut microbiome and glucose metabolism. J Clin Endocrinol Metab.

[177] Salazar J, Angarita L, Morillo V, Navarro C, Martínez MS, Chacín M (2020). Microbiota and diabetes mellitus: role of lipid mediators. Nutrients.

[178] Grasset E, Puel A, Charpentier J, Collet X, Christensen JE, Tercé F (2017). A specific gut microbiota dysbiosis of type 2 diabetic mice induces GLP-1 resistance through an enteric NO-dependent and gut-brain axis mechanism. Cell Metab.

[179] Vrieze A, Van Nood E, Holleman F, Salojärvi J, Kootte RS, Bartelsman JF (2012). Transfer of intestinal microbiota from lean donors increases insulin sensitivity in individuals with metabolic syndrome. Gastroenterology..

[180] Cani PD, Bibiloni R, Knauf C, Waget A, Neyrinck AM, Delzenne NM (2008). Changes in gut microbiota control metabolic endotoxemia-induced inflammation in high-fat diet–induced obesity and diabetes in mice. Diabetes.

[181] Tsiantas K, Konteles SJ, Kritsi E, Sinanoglou VJ, Tsiaka T, Zoumpoulakis P (2022). Effects of non-polar dietary and endogenous lipids on gut microbiota alterations: the role of lipidomics. Int J Mol Sci.

[182] Singh RK, Chang H-W, Yan D, Lee KM, Ucmak D, Wong K (2017). Influence of diet on the gut microbiome and implications for human health. J Transl Med.

[183] Cani PD, Osto M, Geurts L, Everard A (2012). Involvement of gut microbiota in the development of low-grade inflammation and type 2 diabetes associated with obesity. Gut Microbes.

[184] Cândido FG, Valente FX, Grześkowiak ŁM, Moreira APB, Rocha DMUP, Alfenas RdCG (2018). Impact of dietary fat on gut microbiota and low-grade systemic inflammation: mechanisms and clinical implications on obesity. Int J Food Sci Nutr.

[185] Mitchell CM, Davy BM, Halliday TM, Hulver MW, Neilson AP, Ponder MA (2015). The effect of prebiotic supplementation with inulin on cardiometabolic health: rationale, design, and methods of a controlled feeding efficacy trial in adults at risk of type 2 diabetes. Contemp Clin Trials.

[186] Maciejewska D, Skonieczna-Zydecka K, Lukomska A, Gutowska I, Dec K, Kupnicka P (2018). The short chain fatty acids and lipopolysaccharides status in Sprague-Dawley rats fed with high-fat and high-cholesterol diet. J Physiol Pharmacol.

[187] Pearce S, Mani V, Weber T, Rhoads R, Patience J, Baumgard L (2013). Heat stress and reduced plane of nutrition decreases intestinal integrity and function in pigs. J Anim Sci.

[188] Cani PD, Amar J, Iglesias MA, Poggi M, Knauf C, Bastelica D (2007). Metabolic endotoxemia initiates obesity and insulin resistance. Diabetes.

[189] Everard A, Belzer C, Geurts L, Ouwerkerk JP, Druart C, Bindels LB (2013). Cross-talk between *Akkermansia muciniphila* and intestinal epithelium controls diet-induced obesity. Proc Natl Acad Sci.

[190] Ding S, Chi MM, Scull BP, Rigby R, Schwerbrock NM, Magness S (2010). High-fat diet: bacteria interactions promote intestinal inflammation which precedes and correlates with obesity and insulin resistance in mouse. PLoS ONE.

[191] Kim K-A, Gu W, Lee I-A, Joh E-H, Kim D-H (2012). High fat diet-induced gut microbiota exacerbates inflammation and obesity in mice via the TLR4 signaling pathway. PLoS ONE..

[192] Anderson AS, Haynie KR, McMillan RP, Osterberg KL, Boutagy NE, Frisard MI (2015). Early skeletal muscle adaptations to short-term high-fat diet in humans before changes in insulin sensitivity. Obesity.

[193] Harte AL, Varma MC, Tripathi G, McGee KC, Al-Daghri NM, Al-Attas OS (2012). High fat intake leads to acute postprandial exposure to circulating endotoxin in type 2 diabetic subjects. Diabetes Care.

[194] Ghanim H, Abuaysheh S, Sia CL, Korzeniewski K, Chaudhuri A, Fernandez-Real JM (2009). Increase in plasma endotoxin concentrations and the expression of Toll-like receptors and suppressor of cytokine signaling-3 in mononuclear cells after a high-fat, high-carbohydrate meal: implications for insulin resistance. Diabetes Care.

[195] Liang H, Hussey SE, Sanchez-Avila A, Tantiwong P, Musi N (2013). Effect of lipopolysaccharide on inflammation and insulin action in human muscle. PLoS ONE.

[196] Dasu MR, Devaraj S, Park S, Jialal I (2010). Increased toll-like receptor (TLR) activation and TLR ligands in recently diagnosed type 2 diabetic subjects. Diabetes Care.

[197] Shi H, Kokoeva MV, Inouye K, Tzameli I, Yin H, Flier JS (2006). TLR4 links innate immunity and fatty acid–induced insulin resistance. J Clin Investig.

[198] Tsukumo DM, Carvalho-Filho MA, Carvalheira JB, Prada PO, Hirabara SM, Schenka AA (2007). Loss-of-function mutation in Toll-like receptor 4 prevents diet-induced obesity and insulin resistance. Diabetes.

[199] Hulston CJ, Churnside AA, Venables MC (2015). Probiotic supplementation prevents high-fat, overfeeding-induced insulin resistance in human subjects. Br J Nutr.

[200] Foley KP, Denou E, Duggan BM, Chan R, Stearns JC, Schertzer JD (2017). Long-term dysbiosis promotes insulin resistance during obesity despite rapid diet-induced changes in the gut microbiome of mice. BioRxiv..

[201] de La Serre CB, Ellis CL, Lee J, Hartman AL, Rutledge JC, Raybould HE (2010). Propensity to high-fat diet-induced obesity in rats is associated with changes in the gut microbiota and gut inflammation. Am J Physiol Gastrointest Liver Physiol.

[202] Lam YY, Ha CW, Hoffmann JM, Oscarsson J, Dinudom A, Mather TJ (2015). Effects of dietary fat profile on gut permeability and microbiota and their relationships with metabolic changes in mice. Obesity.

[203] Caesar R, Tremaroli V, Kovatcheva-Datchary P, Cani PD, Bäckhed F (2015). Crosstalk between gut microbiota and dietary lipids aggravates WAT inflammation through TLR signaling. Cell Metab.

[204] Just S, Mondot S, Ecker J, Wegner K, Rath E, Gau L (2018). The gut microbiota drives the impact of bile acids and fat source in diet on mouse metabolism. Microbiome.

[205] Fu Y, Wang Y, Gao H, Li D, Jiang R, Ge L (2021). Associations among dietary omega-3 polyunsaturated fatty acids, the gut microbiota, and intestinal immunity. Mediators Inflamm.

[206] Husson M-O, Ley D, Portal C, Gottrand M, Hueso T, Desseyn J-L (2016). Modulation of host defence against bacterial and viral infections by omega-3 polyunsaturated fatty acids. J Infect.

[207] Patterson E, O'Doherty RM, Murphy EF, Wall R, O'Sullivan O, Nilaweera K (2014). Impact of dietary fatty acids on metabolic activity and host intestinal microbiota composition in C57BL/6J mice. Br J Nutr.

[208] Machate DJ, Figueiredo PS, Marcelino G, Guimarães RdCA, Hiane PA, Bogo D (2020). Fatty acid diets: regulation of gut microbiota composition and obesity and its related metabolic dysbiosis. Int J Mol Sci.

[209] Wijekoon MP, Parrish CC, Mansour A (2014). Effect of dietary substitution of fish oil with flaxseed or sunflower oil on muscle fatty acid composition in juvenile steelhead trout (Oncorhynchus mykiss) reared at varying temperatures. Aquaculture.

[210] Ochoa-Repáraz J, Kasper LH (2016). The second brain: is the gut microbiota a link between obesity and central nervous system disorders?. Curr Obes Rep.

[211] Zhu L, Sha L, Li K, Wang Z, Wang T, Li Y (2020). Dietary flaxseed oil rich in omega-3 suppresses severity of type 2 diabetes mellitus via anti-inflammation and modulating gut microbiota in rats. Lipids Health Dis.

[212] Turnbaugh PJ, Bäckhed F, Fulton L, Gordon JI (2008). Diet-induced obesity is linked to marked but reversible alterations in the mouse distal gut microbiome. Cell Host Microbe.

[213] Miao Z, Lin J-s, Mao Y, Chen G-d, Zeng F-f, Dong H-l (2020). Erythrocyte n-6 polyunsaturated fatty acids, gut microbiota, and incident type 2 diabetes: a prospective cohort study. Diabetes Care..

[214] Liu H, Pan L-L, Lv S, Yang Q, Zhang H, Chen W (2019). Alterations of gut microbiota and blood lipidome in gestational diabetes mellitus with hyperlipidemia. Front Physiol.

[215] Lamichhane S, Sen P, Alves MA, Ribeiro HC, Raunioniemi P, Hyötyläinen T (2021). Linking gut microbiome and lipid metabolism: moving beyond associations. Metabolites.

[216] Kim EJ, Ramachandran R, Wierzbicki AS (2022). Lipidomics in diabetes. Curr Opin Endocrinol Diabetes Obes.

[217] Brown EM, Ke X, Hitchcock D, Jeanfavre S, Avila-Pacheco J, Nakata T (2019). Bacteroides-derived sphingolipids are critical for maintaining intestinal homeostasis and symbiosis. Cell Host Microbe.

[218] Holland WL, Bikman BT, Wang L-P, Yuguang G, Sargent KM, Bulchand S (2011). Lipid-induced insulin resistance mediated by the proinflammatory receptor TLR4 requires saturated fatty acid–induced ceramide biosynthesis in mice. J Clin Investig.

[219] Leuti A, Fazio D, Fava M, Piccoli A, Oddi S, Maccarrone M (2020). Bioactive lipids, inflammation and chronic diseases. Adv Drug Deliv Rev.

[220] Chew WS, Torta F, Ji S, Choi H, Begum H, Sim X (2019). Large-scale lipidomics identifies associations between plasma sphingolipids and T2DM incidence. JCI Insight.

[221] Fretts AM, Jensen PN, Hoofnagle AN, McKnight B, Howard BV, Umans J (2021). Plasma ceramides containing saturated fatty acids are associated with risk of type 2 diabetes. J Lipid Res.

[222] Zhang L, Hu Y, An Y, Wang Q, Liu J, Wang G (2022). The Changes of lipidomic profiles reveal therapeutic effects of exenatide in patients with type 2 diabetes. Front Endocrinol.

[223] Floegel A, Stefan N, Yu Z, Mühlenbruch K, Drogan D, Joost H-G (2013). Identification of serum metabolites associated with risk of type 2 diabetes using a targeted metabolomic approach. Diabetes.

[224] Suvitaival T, Bondia-Pons I, Yetukuri L, Pöhö P, Nolan JJ, Hyötyläinen T (2018). Lipidome as a predictive tool in progression to type 2 diabetes in Finnish men. Metabolism.

[225] Yea K, Kim J, Yoon JH, Kwon T, Kim JH, Lee BD (2009). Lysophosphatidylcholine activates adipocyte glucose uptake and lowers blood glucose levels in murine models of diabetes. J Biol Chem.

[226] Prada M, Wittenbecher C, Eichelmann F, Wernitz A, Drouin-Chartier J-P, Schulze MB (2021). Association of the odd-chain fatty acid content in lipid groups with type 2 diabetes risk: a targeted analysis of lipidomics data in the EPIC-Potsdam cohort. Clin Nutr.

[227] Kreznar JH, Keller MP, Traeger LL, Rabaglia ME, Schueler KL, Stapleton DS (2017). Host genotype and gut microbiome modulate insulin secretion and diet-induced metabolic phenotypes. Cell Rep.

[228] Haeusler RA, Astiarraga B, Camastra S, Accili D, Ferrannini E (2013). Human insulin resistance is associated with increased plasma levels of 12α-hydroxylated bile acids. Diabetes.

[229] Wewalka M, Patti M-E, Barbato C, Houten SM, Goldfine AB (2014). Fasting serum taurine-conjugated bile acids are elevated in type 2 diabetes and do not change with intensification of insulin. J Clin Endocrinol Metab.

[230] Ryan KK, Tremaroli V, Clemmensen C, Kovatcheva-Datchary P, Myronovych A, Karns R (2014). FXR is a molecular target for the effects of vertical sleeve gastrectomy. Nature.

[231] Islam KS, Fukiya S, Hagio M, Fujii N, Ishizuka S, Ooka T (2011). Bile acid is a host factor that regulates the composition of the cecal microbiota in rats. Gastroenterology.

[232] Zhang S-Y, Li RJ, Lim Y-M, Batchuluun B, Liu H, Waise TZ (2021). FXR in the dorsal vagal complex is sufficient and necessary for upper small intestinal microbiome-mediated changes of TCDCA to alter insulin action in rats. Gut.

[233] Muccioli GG, Naslain D, Bäckhed F, Reigstad CS, Lambert DM, Delzenne NM (2010). The endocannabinoid system links gut microbiota to adipogenesis. Mol Syst Biol.

[234] Lynch A, Crowley E, Casey E, Cano R, Shanahan R, McGlacken G (2017). The Bacteroidales produce an N-acylated derivative of glycine with both cholesterol-solubilising and hemolytic activity. Sci Rep.

[235] Boutagy NE, McMillan RP, Frisard MI, Hulver MW (2016). Metabolic endotoxemia with obesity: is it real and is it relevant?. Biochimie.

[236] Geurts L, Everard A, Van Hul M, Essaghir A, Duparc T, Matamoros S (2015). Adipose tissue NAPE-PLD controls fat mass development by altering the browning process and gut microbiota. Nat Commun.

[237] Leylabadlo HE, Sanaie S, Heravi FS, Ahmadian Z, Ghotaslou R (2020). From role of gut microbiota to microbial-based therapies in type 2-diabetes. Infect Genet Evol.

[238] Ojo O, Ojo OO, Zand N, Wang X (2021). The effect of dietary fibre on gut microbiota, lipid profile, and inflammatory markers in patients with type 2 diabetes: a systematic review and meta-analysis of randomised controlled trials. Nutrients.

[239] Fallucca F, Porrata C, Fallucca S, Pianesi M (2014). Influence of diet on gut microbiota, inflammation and type 2 diabetes mellitus. First experience with macrobiotic Ma-Pi 2 diet. Diabetes Metabol Res Rev.

[240] Ibanez C, Mouhid L, Reglero G, Ramirez de Molina A (2017). Lipidomics insights in health and nutritional intervention studies. J Agric Food Chem.

[241] Zhang N, Ju Z, Zuo T (2018). Time for food: The impact of diet on gut microbiota and human health. Nutrition.

[242] McKenney PT, Pamer EG (2015). From hype to hope: the gut microbiota in enteric infectious disease. Cell.

[243] Clark A, Mach N (2016). Exercise-induced stress behavior, gut-microbiota-brain axis and diet: a systematic review for athletes. J Int Soc Sports Nutr.

[244] Yang Q, Liang Q, Balakrishnan B, Belobrajdic DP, Feng Q-J, Zhang W (2020). Role of dietary nutrients in the modulation of gut microbiota: a narrative review. Nutrients.

[245] Stacchiotti V, Rezzi S, Eggersdorfer M, Galli F (2021). Metabolic and functional interplay between gut microbiota and fat-soluble vitamins. Crit Rev Food Sci Nutr.

[246] Ye Z, Xu Y-J, Liu Y (2021). Influences of dietary oils and fats, and the accompanied minor content of components on the gut microbiota and gut inflammation: a review. Trends Food Sci Technol.

[247] Kootte R, Vrieze A, Holleman F, Dallinga-Thie GM, Zoetendal EG, de Vos WM (2012). The therapeutic potential of manipulating gut microbiota in obesity and type 2 diabetes mellitus. Diabetes Obes Metab.

[248] Kumar M, Pal N, Sharma P, Kumawat M, Sarma DK, Nabi B (2022). Omega-3 fatty acids and their interaction with the gut microbiome in the prevention and amelioration of type-2 diabetes. Nutrients.

[249] Sagild U, Littauer J, Jespersen CS, Andersen S (1965). Epidemiological studies in Greenland 1962–1964. 1. Diabetes mellitus in Eskimos. Acta Medica Scandinavica..

[250] Salas-Salvadó J, Bulló M, Babio N, Martínez-González MÁ, Ibarrola-Jurado N, Basora J (2011). Reduction in the incidence of type 2 diabetes with the Mediterranean diet: results of the PREDIMED-Reus nutrition intervention randomized trial. Diabetes Care.

[251] Iwase Y, Kamei N, Takeda-Morishita M (2015). Antidiabetic effects of omega-3 polyunsaturated fatty acids: from mechanism to therapeutic possibilities. Pharmacol Pharm.

[252] Shama S, Liu W (2020). Omega-3 fatty acids and gut microbiota: a reciprocal interaction in nonalcoholic fatty liver disease. Dig Dis Sci.

[253] Silva YP, Bernardi A, Frozza RL (2020). The role of short-chain fatty acids from gut microbiota in gut-brain communication. Front Endocrinol.

[254] Salamone D, Rivellese AA, Vetrani C (2021). The relationship between gut microbiota, short-chain fatty acids and type 2 diabetes mellitus: the possible role of dietary fibre. Acta Diabetol.

[255] Yan M, Cai WB, Hua T, Cheng Q, Ai D, Jiang HF (2020). Lipidomics reveals the dynamics of lipid profile altered by omega-3 polyunsaturated fatty acid supplementation in healthy people. Clin Exp Pharmacol Physiol.

[256] Peng W, Huang J, Yang J, Zhang Z, Yu R, Fayyaz S (2020). Integrated 16S rRNA sequencing, metagenomics, and metabolomics to characterize gut microbial composition, function, and fecal metabolic phenotype in non-obese type 2 diabetic Goto-Kakizaki rats. Front Microbiol.

[257] Wang DD (2018). Dietary n-6 polyunsaturated fatty acids and cardiovascular disease: Epidemiologic evidence. Prostaglandins Leukot Essent Fatty Acids.

[258] Tortosa-Caparrós E, Navas-Carrillo D, Marín F, Orenes-Piñero E (2017). Anti-inflammatory effects of omega 3 and omega 6 polyunsaturated fatty acids in cardiovascular disease and metabolic syndrome. Crit Rev Food Sci Nutr.

[259] Innes JK, Calder PC (2018). Omega-6 fatty acids and inflammation. Prostaglandins Leukot Essent Fatty Acids.

[260] Ferrer R, Moreno JJ (2010). Role of eicosanoids on intestinal epithelial homeostasis. Biochem Pharmacol.

[261] An J-U, Hong S-H, Oh D-K (2018). Regiospecificity of a novel bacterial lipoxygenase from *Myxococcus xanthus* for polyunsaturated fatty acids. Biochim Biophys Acta Mol Cell Biol Lipids..

[262] Luo P, Wang M-H (2011). Eicosanoids, β-cell function, and diabetes. Prostaglandins Other Lipid Mediat.

[263] Brzeska M, Szymczyk K, Szterk A (2016). Current knowledge about oxysterols: a review. J Food Sci.

[264] Cuevas-Tena M, Alegría A, Lagarda MJ (2018). Relationship between dietary sterols and gut microbiota: a review. Eur J Lipid Sci Technol.

[265] Hovenkamp E, Demonty I, Plat J, Lütjohann D, Mensink RP, Trautwein EA (2008). Biological effects of oxidized phytosterols: a review of the current knowledge. Prog Lipid Res.

[266] Quifer-Rada P, Choy YY, Calvert CC, Waterhouse AL, Lamuela-Raventos RM (2016). Use of metabolomics and lipidomics to evaluate the hypocholestreolemic effect of Proanthocyanidins from grape seed in a pig model. Mol Nutr Food Res.

[267] Cuevas-Tena M, Alegria A, Lagarda MJ, Venema K (2019). Impact of plant sterols enrichment dose on gut microbiota from lean and obese subjects using TIM-2 in vitro fermentation model. J Funct Foods.

[268] Cuevas-Tena M, Bermúdez JD, de los Ángeles Silvestre R, Alegría A, Lagarda MJ (2019). Impact of colonic fermentation on sterols after the intake of a plant sterol-enriched beverage: a randomized, double-blind crossover trial. Clin Nutr.

[269] Weststrate J, Ayesh R, Bauer-Plank C, Drewitt P (1999). Safety evaluation of phytosterol esters. Part 4. Faecal concentrations of bile acids and neutral sterols in healthy normolipidaemic volunteers consuming a controlled diet either with or without a phytosterol ester-enriched margarine. Food Chem Toxicol.

[270] Strandberg TE, Tilvis RS, Pitkala KH, Miettinen TA (2006). Cholesterol and glucose metabolism and recurrent cardiovascular events among the elderly: a prospective study. J Am Coll Cardiol.

[271] Strandberg TE, Salomaa V, Venhanen H, Miettinen TA (1996). Associations of fasting blood glucose with cholesterol absorption and synthesis in nondiabetic middle-aged men. Diabetes.

[272] Hallikainen M, Toppinen L, Mykkänen H, Ågren JJ, Laaksonen DE, Miettinen TA (2006). Interaction between cholesterol and glucose metabolism during dietary carbohydrate modification in subjects with the metabolic syndrome. Am J Clin Nutr.

[273] Blanco-Morales V, Garcia-Llatas G, MaJ Yebra, Sentandreu V, Lagarda MJ, Alegría A (2019). Impact of a plant sterol-and galactooligosaccharide-enriched beverage on colonic metabolism and gut microbiota composition using an in vitro dynamic model. J Agric Food Chem.

[274] Xiao L, Cui T, Liu S, Chen B, Wang Y, Yang T (2019). Vitamin A supplementation improves the intestinal mucosal barrier and facilitates the expression of tight junction proteins in rats with diarrhea. Nutrition.

[275] Tian Y, Nichols RG, Cai J, Patterson AD, Cantorna MT (2018). Vitamin A deficiency in mice alters host and gut microbial metabolism leading to altered energy homeostasis. J Nutr Biochem.

[276] Hibberd MC, Wu M, Rodionov DA, Li X, Cheng J, Griffin NW (2017). The effects of micronutrient deficiencies on bacterial species from the human gut microbiota. Sci Transl Med.

[277] Lv Z, Wang Y, Yang T, Zhan X, Li Z, Hu H (2016). Vitamin A deficiency impacts the structural segregation of gut microbiota in children with persistent diarrhea. J Clin Biochem Nutr.

[278] Silvagno F, Pescarmona G (2017). Spotlight on vitamin D receptor, lipid metabolism and mitochondria: Some preliminary emerging issues. Mol Cell Endocrinol.

[279] Sacerdote A, Dave P, Lokshin V, Bahtiyar G (2019). Type 2 diabetes mellitus, insulin resistance, and vitamin D. Curr DiabRep.

[280] Fleet JC (2017). The role of vitamin D in the endocrinology controlling calcium homeostasis. Mol Cell Endocrinol.

[281] Thomas RL, Jiang L, Adams JS, Xu ZZ, Shen J, Janssen S (2020). Vitamin D metabolites and the gut microbiome in older men. Nat Commun.

[282] Charoenngam N, Holick MF (2020). Immunologic effects of vitamin D on human health and disease. Nutrients.

[283] Singh P, Rawat A, Alwakeel M, Sharif E, Al KS (2020). The potential role of vitamin D supplementation as a gut microbiota modifier in healthy individuals. Sci Rep.

[284] Haro C, Montes-Borrego M, Rangel-Zúñiga OA, Alcalá-Díaz JF, Gómez-Delgado F, Pérez-Martínez P (2016). Two healthy diets modulate gut microbial community improving insulin sensitivity in a human obese population. J Clin Endocrinol.

[285] Liu J, Wu S, Cheng Y, Liu Q, Su L, Yang Y (2021). Sargassum fusiforme alginate relieves hyperglycemia and modulates intestinal microbiota and metabolites in type 2 diabetic mice. Nutrients.

[286] Choi Y, Lee S, Kim S, Lee J, Ha J, Oh H (2020). Vitamin E (α-tocopherol) consumption influences gut microbiota composition. Int J Food Sci Nutr.

[287] Maggini S, Wintergerst ES, Beveridge S, Hornig DH (2007). Selected vitamins and trace elements support immune function by strengthening epithelial barriers and cellular and humoral immune responses. Br J Nutr.

[288] Rinninella E, Mele MC, Merendino N, Cintoni M, Anselmi G, Caporossi A (2018). The role of diet, micronutrients and the gut microbiota in age-related macular degeneration: new perspectives from the gut–retina axis. Nutrients.

[289] Mandal S, Godfrey KM, McDonald D, Treuren WV, Bjørnholt JV, Midtvedt T (2016). Fat and vitamin intakes during pregnancy have stronger relations with a pro-inflammatory maternal microbiota than does carbohydrate intake. Microbiome.

[290] Tang M, Frank DN, Sherlock L, Ir D, Robertson CE, Krebs NF (2016). Effect of vitamin E with therapeutic iron supplementation on iron repletion and gut microbiome in US iron deficient infants and toddlers. J Pediatr Gastroenterol Nutr.

[291] Yang C, Zhao Y, Im S, Nakatsu C, Jones-Hall Y, Jiang Q (2021). Vitamin E delta-tocotrienol and metabolite 13’-carboxychromanol inhibit colitis-associated colon tumorigenesis and modulate gut microbiota in mice. J Nutr Biochem.

[292] Naziroğlu M, Güler T, Yüce A (2002). Effect of vitamin E on ruminal fermentation in vitro. J Vet Med Ser A.

[293] Wei C, Lin S, Wu J, Zhao G, Zhang T, Zheng W (2015). Effects of supplementing vitamin E on in vitro rumen gas production, volatile fatty acid production, dry matter disappearance rate, and utilizable crude protein. Czeh J Anim Sci.

[294] Manna P, Kalita J (2016). Beneficial role of vitamin K supplementation on insulin sensitivity, glucose metabolism, and the reduced risk of type 2 diabetes: a review. Nutrition.

[295] Shearer MJ, Fu X, Booth SL (2012). Vitamin K nutrition, metabolism, and requirements: current concepts and future research. Adv Nutr.

[296] Li Y, peng Chen J, Duan L, Li S. (2018). Effect of vitamin K2 on type 2 diabetes mellitus: a review. Diabetes Res Clin Pract.

[297] Ferland G (2012). Vitamin K and the nervous system: an overview of its actions. Adv Nutr.

[298] Lev M, Milford A (1972). Effect of vitamin K depletion and restoration on sphingolipid metabolism in *Bacteroides melaninogenicus*. J Lipid Res.

[299] Khan SR, Manialawy Y, Obersterescu A, Cox BJ, Gunderson EP, Wheeler MB (2020). Diminished sphingolipid metabolism, a hallmark of future type 2 diabetes pathogenesis, is linked to pancreatic β cell dysfunction. IScience.

[300] Roszczyc-Owsiejczuk K, Zabielski P (2021). Sphingolipids as a culprit of mitochondrial dysfunction in insulin resistance and type 2 diabetes. Front Endocrinol.

[301] Russo S, Ross J, Cowart L (2013). Sphingolipids in obesity, type 2 diabetes, and metabolic disease. Sphingolipids Dis.

[302] Zhang Y, Zhang H (2013). Microbiota associated with type 2 diabetes and its related complications. Food Sci Human Wellness.

[303] Ellis JL, Karl JP, Oliverio AM, Fu X, Soares JW, Wolfe BE (2021). Dietary vitamin K is remodeled by gut microbiota and influences community composition. Gut Microbes.

[304] Rohrhofer J, Zwirzitz B, Selberherr E, Untersmayr E (2021). The impact of dietary sphingolipids on intestinal microbiota and gastrointestinal immune homeostasis. Front Immunol.

[305] Vesper H, Schmelz E-M, Nikolova-Karakashian MN, Dillehay DL, Lynch DV, Merrill AH (1999). Sphingolipids in food and the emerging importance of sphingolipids to nutrition. J Nutr.

[306] Liu Z, Rochfort S, Cocks B (2018). Milk lipidomics: what we know and what we don't. Prog Lipid Res.

[307] Wolters M, Ahrens J, Romaní-Pérez M, Watkins C, Sanz Y, Benítez-Páez A (2019). Dietary fat, the gut microbiota, and metabolic health—a systematic review conducted within the MyNewGut project. Clin Nutr..

